# Environment-induced changes in selective constraints on social learning during the peopling of the Americas

**DOI:** 10.1038/srep44431

**Published:** 2017-03-16

**Authors:** Briggs Buchanan, Anne Chao, Chun-Huo Chiu, Robert K. Colwell, Michael J. O’Brien, Angelia Werner, Metin I. Eren

**Affiliations:** 1 Department of Anthropology, University of Tulsa, Tulsa, OK, 74104, USA; 2 Institute of Statistics, National Tsing Hua University, Hsin-Chu, 30043, Taiwan; 3 Department of Ecology and Evolutionary Biology, University of Connecticut, Storrs, CT, 06269-3043, USA; 4 Museum of Natural History, University of Colorado, Boulder, CO, 80309 USA; 5 Department of History, Texas A&M–San Antonio, San Antonio, TX, USA; 6 Department of Anthropology, University of Missouri, Columbia, MO, 65211, USA; 7 Department of Anthropology, Kent State University, Kent, OH, 44242, USA; 8 Department of Archaeology, Cleveland Museum of Natural History, Cleveland, OH, 44106, USA

## Abstract

The weaponry technology associated with Clovis and related Early Paleoindians represents the earliest well-defined evidence of humans in Pleistocene North America. We assess the technological diversity of these fluted stone points found at archaeological sites in the western and eastern halves of North America by employing statistical tools used in the quantification of ecological biodiversity. Our results demonstrate that the earliest hunters in the environmentally heterogeneous East used a more diverse set of points than those in the environmentally homogenous West. This and other evidence shows that environmental heterogeneity in the East promoted the relaxation of selective constraints on social learning and increased experimentation with point designs.

The Americas were the last major land masses colonized by *Homo sapiens* in their dispersal out of Africa and subsequent spread across the globe^1^. Current evidence indicates that human populations arrived in North America during the terminal Pleistocene^2^,^3^. After about 13,500–13,400 calBP (calendar years before present), the archaeological record in North America is characterized by tools attributed to the Clovis and related Early Paleoindian (EP) cultures^4^,^5^, the most diagnostic of which are fluted stone projectile points (Fig. 1). The rapid geographic expansion of EP groups across North America^3^,^4^,^6^, as well as the technology, mobility patterns, resource procurement, and site size that characterize them, offers evidence of a continental human dispersal event unrestricted by earlier peoples^4^,^7^,^8^. However, given the lack of fluted projectile points in the Old World, the fluted weapon tips probably emerged in the New World, suggesting the existence of at least a small pre-EP population, responsible for innovating “the first American invention” before carrying it across the continent^2^,^4^.

The EP dispersal is exceptional in terms of Pleistocene human migration because, unlike in much of the Old World, the Americas never hosted hominins other than *Homo sapiens*, ameliorating the problem of distinguishing between sites of autochthonous versus invasive populations. Further, given that the EP expansion occurred within a period of less than a millennium, this event benefits from a temporal resolution not currently available in the study of *H. sapiens* dispersals in Africa, Eurasia, or Australia, which occurred tens of millennia earlier and over much longer periods. Thus, while studies of human dispersals in the Old World can provide a broader, long-term perspective, the North American EP dispersal facilitates, on a finer scale, an examination of Stone Age colonization processes and how they played out on a vast, uninhabited continent. One of the primary sources of information about the North American colonization process comes from the study of the period’s distinctive stone points, which have yielded insights into migration routes, mobility, economics, weapon systems, site-location preferences, hunting and domestic activities, cultural evolutionary dynamics, and transmission of technical knowledge^9^,^10^,^11^,^12^,^13^.

## Measuring EP diversity

Measures of diversity are fundamental to the investigation of large-scale patterns in the natural and cultural worlds. Diversity measures have been used to examine patterns in the distribution of species^14^, languages^15^, genes^16^, and technology^17^. In archaeology, statistical measures of diversity have recently begun to be used to explore patterns in assemblages and artifacts over space and time^18^, while intuitive perceptions of stone-point diversity of North America’s earliest Paleoindians have long been used to address questions regarding their origins and adaptations^19^,^20^.

Here we estimate the diversity in EP point form across North America by analyzing a continent-wide dataset with rigorous statistical tools developed for, and widely used by, ecologists to quantify biodiversity. Our sample of complete EP points comes from unmixed assemblages that have been reliably dated to this period, meaning that an assemblage was either associated with radiometric dates in the ca. 13,500–12,800 calBP range in the West and ca. 12,800–12,500 calBP range in the East^3^,^6^,^7^,^21^,^22^,^23^ or contained diagnostic artifacts that are radiometrically dated to these ranges at another site. We used different age ranges for the EP period in the West and East because it appears to be time-transgressive, in that a diffusion process began in the West around 13,500 calBP and ended in the Northeast just after 12,500 calBP^6^,^23^. We restricted our point sample to assemblages, rather than including isolated points, in order to incorporate typical levels of variation. We did this because points in assemblages may fall outside the range of “classic” EP forms, but their presence in an assemblage means they were manufactured by EP tool makers, nonetheless.

**Figure 2:**
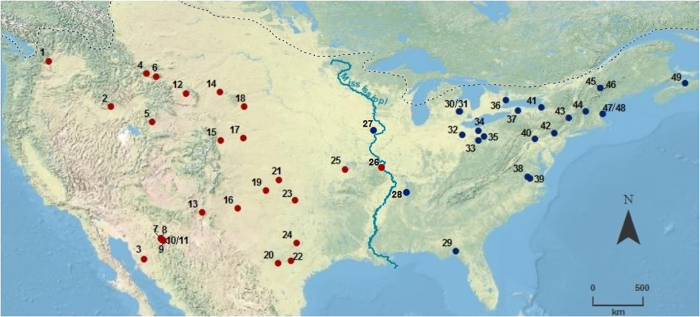
Map of North America showing the location of Clovis assemblages used in the analysis (basemap created in ArcGIS ESRI, http://www.esri.com/). The dashed line shows the extent of glacial ice at approximately 13,300 calBP based on Dyke’s reconstruction (Dyke, A. S. An outline of North American deglaciation with emphasis on central and northern Canada. *Dev. Quat. Sci.* **2**, 373–424 (2004)). (red = West, blue = East; key: 1, East Wenatchee; 2, Simon; 3, El Fin del Mundo; 4, Indian Creek; 5, Fenn; 6, Anzick; 7, Murray Springs; 8, Escapule; 9, Lehner; 10, Naco; 11, Leikum; 12, Colby; 13, Mockingbird Gap; 14, Crook County; 15, Dent; 16, Blackwater Draw; 17, Drake; 18, Lange Ferguson; 19, Miami; 20, Kincaid; 21, Jake Bluff; 22, Pavo Real; 23, Domebo; 24, Gault; 25, Big Eddy; 26, Kimmswick; 27, Rummells-Maske; 28, Carson-Conn-Short; 29, Sloth Hole; 30, Gainey; 31, Butler; 32, Sheriden; 33, Welling; 34, Paleo Crossing; 35, Nobles Pond; 36, Udora; 37, Lamb; 38, Williamson; 39, Cactus Hill; 40, Shoop; 41, Potts; 42, Shawnee–Minisink; 43, West Athens Hill; 44, Whipple; 45, Adkins; 46, Vail; 47, Bull Brook II; 48, Bull Brook; 49, Debert).

The distribution of assemblages in our sample covers most of North America (Fig. 2). Of the assemblages, 26 (with 154 points) are from western North America and 23 (with 138 points) from eastern North America (Supplementary Table 1; classes for the 292 points in the sample are provided in Supplementary Data File). These two regions were defined by their location relative to the Mississippi River. We should note that two sites (Rummells–Maske and Kimmswick) both lie within the Mississippi River drainage and therefore are located in the border area for the division between the western and eastern regions. However, Rummells–Maske is located within the greater Great Lakes region and is commonly considered part of the eastern region, whereas Kimmswick is more traditionally considered a western assemblage.

To investigate diversity we defined fully replicable and explicit classes of EP points using a paradigmatic classification comprising quantitative characters and character states (see Methods). We compared the diversity of points in western versus eastern North America as well as the extent of variation in abundance among classes. To evaluate class diversity, we employed three widely used measures: richness (the number of classes), Shannon diversity (the effective number of common classes), and Simpson diversity (the effective number of dominant classes)^24^. Because both sample size and sample completeness differ between the two regions, and measures of diversity are dependent on sample size and coverage, we used sample-size-based rarefaction and extrapolation^25^ and coverage-based rarefaction and extrapolation^24^ techniques to standardize sample size and sample completeness, thus rendering samples comparable (see Methods). In addition, we estimated asymptotic class diversity for each measure and each region. Heterogeneity was assessed by the coefficient of variation—the ratio of the standard deviation to the mean of abundance among class frequencies.

## Results

**Figure 3:**
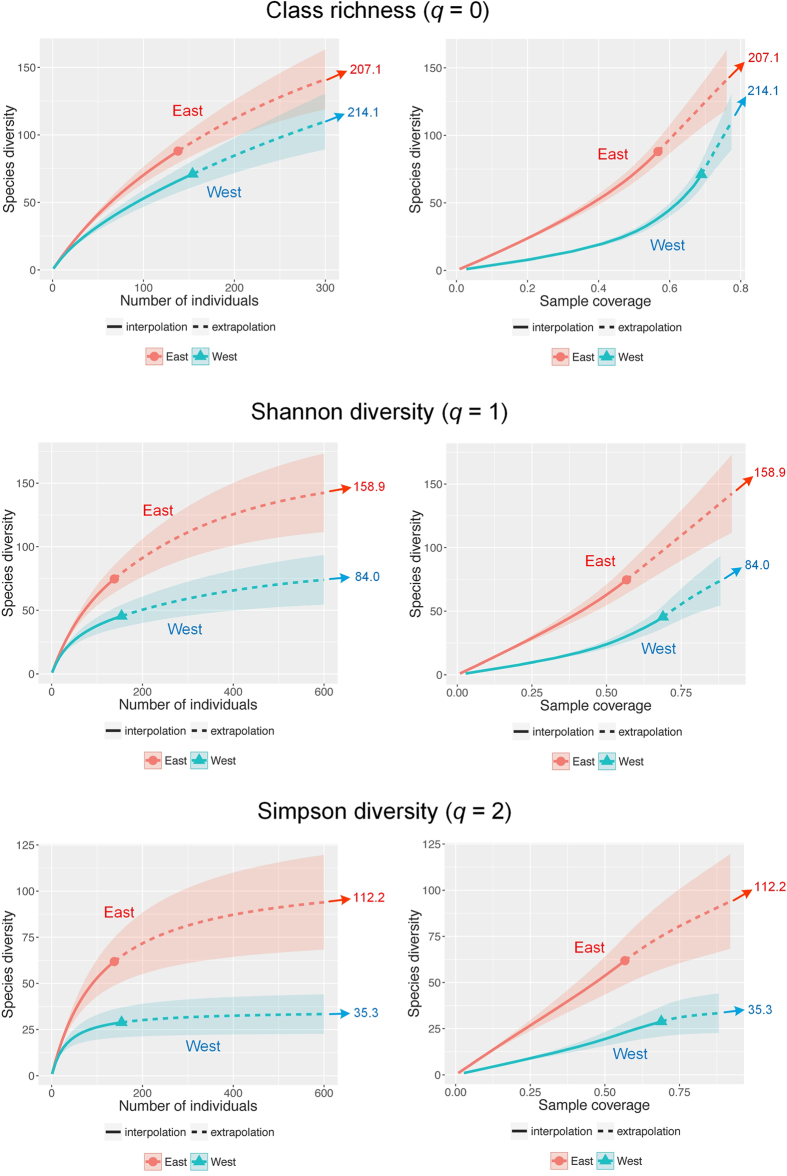
Comparison of sample-size-based (left panels) and sample-coverage-based (right panels) rarefaction and extrapolation for class richness (upper panels), Shannon diversity (middle panels) and Simpson diversity (lower panels) for Clovis points from the eastern and western United States. Observed samples are denoted by solid dots; rarefied segments are shown by solid lines and extrapolated segments by broken lines. The extrapolation extends up to a base size of 300 points for class richness and to a base size of 600 for Shannon diversity and Simpson diversity. The sample-coverage-based extrapolation for class richness extends to the coverage value of the corresponding maximum size of 300 (76.0% for the East and 77.4% for the West). For Shannon and Simpson diversities, the extrapolation is extended to the coverage value of the corresponding maximum size of 600 (92.0% for the East and 88.2% for the West). The 95% confidence intervals (shaded areas) were obtained by a bootstrap method based on 200 replications. The estimated asymptote (given in Table 1) of diversity for each curve is displayed next to the arrow at the right-hand end of each curve.

Points from the West (n = 154 points) were placed in 71 classes and points from the East (n = 138) in 88 classes. Sample completeness (as measured by sample coverage, see Methods) was 56.7% in the East and 68.9% in the West. The results of our diversity analyses appear in Table 1 and Fig. 3, the latter of which shows that, for any standardized sample size and any standardized sample completeness, point diversity in the East is consistently higher than in the West, regardless of diversity measure. This conclusion is also valid for all three asymptotic richness measures (Table 1). The 95% confidence intervals for the two areas do not overlap, either for Shannon diversity (common point classes) or for Simpson diversity (dominant point classes), implying that the East is significantly more diverse than the West for these measures (middle and lower rows of Fig. 3; Table 1b and c). For class richness, the same pattern is significant up to the 80% fraction of the assemblages, but significant difference in richness cannot be demonstrated for larger fractions due to undetected classes (upper right row of Fig. 3).

The heterogeneity of point classes in the West (CV = 1.387) is significantly greater than in the East (CV = 0.632) (Table 1). In other words, in the West some point forms are quite abundant, and other forms are relatively rare. In the East, in contrast, class abundance is much more even, with point forms in the sample less variable in frequency.

Our findings that the East has a richer suite of EP point classes than the West, although point classes are more heterogeneous in frequency in the West, proved robust to several analyses that altered the scope of the data for the computations reported above. First, we iteratively removed the largest and potentially most influential assemblages from the East and West (Supplementary Figs 2 to 7; Supplementary Tables 2 to 7). The six assemblages include four assemblages in the East (Bull Brook, Vail, Shoop and Gainey) and two assemblages in the West (Mockingbird Gap and Drake). The results of these analyses yielded similar outcomes: the East has a richer set of point classes than the West. Second, we iteratively removed each of the seven characters (Supplementary Figs 8 to 14; Supplementary Tables 8 to 14). The results of these analyses also yielded similar outcomes as above, showing that no single site and no single character had a disproportionate impact on the results. Lastly, we altered the breadth of character states of characters II, III, IV, and VII (Supplementary Fig. 15; Supplementary Table 15). The character states for these characters were defined by specifying ranges within continuous variables, thus lacking a “multistate” option. The assessments of richness and heterogeneity for these analyses again remained qualitatively similar to those presented above.

## Discussion

We offer three explanations for the significant, consistent, and robust differences in diversity of Clovis and related Early Paleoindian point forms between regions. First, the pattern of differences could indicate that the geographic origin of the EP dispersal lies in eastern North America, a hypothetically deeper time in this region accounting for proliferation of variation, expressed by greater richness and lower heterogeneity of point-class frequencies^19^,^20^. However, this conjecture is inconsistent with current radiocarbon and ancient genetic evidence, which, considered together, strongly indicate a western rather than eastern origin for Paleoindians. The current best radiocarbon estimates for the age of Clovis indicate that the West was colonized well before the East^3^,^6^,^7^,^21^,^22^,^23^. Finally, ancient genetic evidence extracted from skeletal remains of a Pleistocene-age infant from Montana associated with Clovis artifacts shows a pattern of ancestry that fits with a migration route leading from northeastern Asia to northwestern North America^26^.

A second possible explanation is that increased population fissioning and subsequent isolation of EP groups in the East led to the creation of the greater richness and heterogeneity of point-class frequencies in the East compared to in the West. Analytical and empirical evidence supports the notion that technology used by groups in isolation tends to drift as a result of sampling effects of the initial split (founder effect) and from the accumulation of subsequent innovations and mutations introduced during manufacture^27^,^28^,^29^. Although it is likely that these processes played a role in creating novel point classes^30^, there is no evidence to suggest that cultural fissioning and isolation were occurring at a higher rate in the East than in the West. On the contrary, a network analysis of stone raw materials used by Early Paleoindians across the continent demonstrated that eastern North America possessed the largest network, whereas western North America contained two smaller networks^31^.

**Figure 4:**
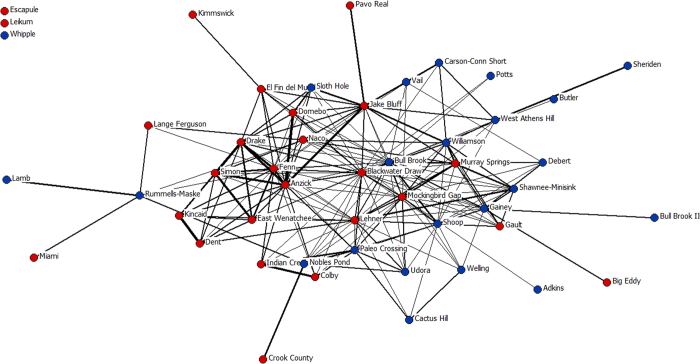
Spring-embedded network map of shared Clovis point classes among assemblages. Assemblages are nodes color-coded by macroregion (red = West, blue = East). Ties are based on shared point classes among assemblages and are weighted using the Sørensen Index.

To further evaluate the possibility that EP groups in the East were more isolated than those in the West, we constructed a network of shared point classes among assemblages. The network of shared point classes reveals some clustering by region, but 26 point classes are shared among assemblages in the West and East (Fig. 4, see Methods for details). Most assemblages form a large network of shared classes, and only three assemblages do not share point classes with any other assemblage (two in the West and one in the East). The interconnectedness of the large network suggests that group fissioning and subsequent isolation is an unlikely source of the diversity differences between the West and the East.

A third, and currently most plausible, explanation for the diversity is that environmental differences between these two broad regions (see below) promoted the relaxation of selective constraints for social learning with respect to stone-point technology. The lower richness and greater heterogeneity of point classes in the West is consistent with a stronger degree of social learning relative to the East, as western groups produced fewer point forms overall and a few particular forms were produced more frequently. Moreover, the presence in the West of fewer point forms, but in greater abundance, also suggests more strongly biased transmission in the West relative to in the East, which may have been acting to limit variation (see below), as has been proposed for the prehistoric Great Basin^32^.

Current evidence for the nature of late Pleistocene environments in the East and in the West suggests substantial differences. Cannon and Meltzer^33^ note that relative to western terminal Pleistocene environments, contemporaneous environments in the East were vegetationally and faunally more heterogeneous, incorporating a greater number of habitat types within any area of a given size, and thus possessing greater variability in the size and distribution of resource patches available to humans. In the more heterogeneous environment of the East, human foragers are expected to have dispersed and exploited numerous and varied prey patches^33^,^34^, corroborated by EP sites in eastern North America exhibiting richer faunal assemblages relative to those in the West.

Thus, as groups moved from west to east across the continent, a shift in subsistence strategy would have come with a substantial cost to forager time budgets—a factor demonstrated experimentally, ethnographically, and through simulations to significantly influence variation in artifacts and toolkits^35^. Relative to their western kin, colonizers of the East would have had to invest more time in accumulating the knowledge needed to exploit a greater variety of prey patches, in learning an unfamiliar landscape to understand where productive resource patches could be located, and in traveling between a greater number of smaller patches. Time available for teaching and learning stone-point production in eastern groups would have been comparatively diminished, leading to a concomitant flourish of individual trial-and-error learning and experimentation, resulting in increased tool “mutation rates”^35^. Conversely, Clovis-point production in the West appears to have been more specialized, perhaps as a result of the focus on fewer prey species in a more stable environment. Previous studies that have shown differences between Clovis points used on mammoth versus those used to hunt bison in the West provide some support for this proposition^36^.

In this way, point-class richness may have increased and point-class heterogeneity decreased among eastern groups, relative to western groups. Not only would the time available for teaching and learning be less in the East, but also accumulated cultural knowledge may have become relatively less useful in the East^37^, as the match between environment and technology became increasingly discordant^38^. Thus, it is likely that the heterogeneous environments in the East diminished the benefits of biased social learning, whereas individuals relying on relatively more trial-and-error experimentation with point forms at the colonizing front would have been more successful^38^—a pattern noted elsewhere^39^.

For more than 50 years the geographic distribution of EP point diversity and the cause of that diversity have been debated. Traditionally, the area of greatest known point-class richness—the East—has been equated with the “origin” of the EP dispersal, whereas class heterogeneity has been ignored. However, our statistical assessments of EP point diversity, when considered with other multidisciplinary data and analyses, suggest a new model. We suggest that quantitative patterns of point diversity indicate that environmental differences played a significant role in altering the learning systems of North America’s earliest inhabitants as they expanded eastward across the continent from primarily social (biased) learning in the West to mostly individual learning in the East. This inference, in turn, contributes to our understanding of why *H. sapiens* was ultimately successful in peopling the globe. Given the speed with which the colonization of North America occurred, we can infer that within a small number of generations groups had the flexibility to modify learning systems, from primarily social to primarily, although not exclusively, individual learning, maximizing their chances of success as they dispersed into new and unfamiliar landscapes.

### Materials return

#### Paradigmatic Classification and Class Construction

Paradigmatic classification is a dimensional classification procedure in which the units, i.e. classes, are defined by intersection, with each dimension (henceforth character) treated as a set of mutually exclusive alternate features (henceforth character states)^38^,^40^,^41^,^42^. All character states belonging to a single character share the ability to combine, in principle, with character states of each other character. Dunnell^40^ specified, “In paradigmatic classification all of the class definitions are drawn from the same set of dimensions [characters] of features [character states]. Individual classes are distinguished from one another by the unique product obtained in the combination, permutation, or intersection of features [character states] from the set of dimensions [characters].”

Dunnell^40^ noted that paradigmatic classes possess three important properties, given their creation by the intersection of character states. First, all of the characters and character states are equivalent; none is or can be weighted more or less than any other. Second, paradigmatic classes are unambiguous, given that character states within a single character are mutually exclusive, and the intersection of character states from different characters prevents internal contradiction. Third, paradigmatic classes are comparable; that is, one class is comparable with all other classes in the same classification. In other words, “the structure of paradigmatic classification always specifies that all classes within it differ from one another in the same manner”^39^. For these reasons, paradigmatic classification is an ideal approach to assessing the diversity of archaeological material culture^42^. Moreover, this approach responds to calls by archaeologists for stone point analytical units to be explicit and to proceed independently from traditional typological biases and preconceptions^43^,^44^.

To investigate EP point diversity, we created fully replicable and explicit classes of EP points using a paradigmatic classification. Our classification builds on the classification of O’Brien *et al*.^38^ (see also^42^,^45^), but differs in that it is structured entirely on quantitative characters and character states. These characters and characters states are:

Character I. Location of Maximum Blade Width.

**Figure 5:**
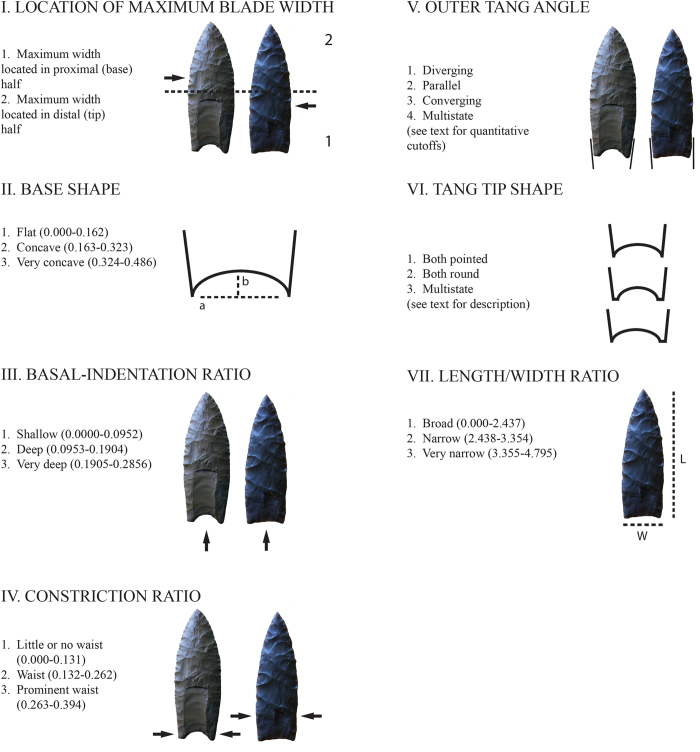
Characters and character states for the point paradigmatic classification.

This character was assessed by dividing a point, transversely, into halves and measuring which half possessed the larger width (Fig. 5). This division yielded two character states:

1. Maximum width located in proximal (base) half
2. Maximum width located in distal (tip) half

Character II. Base Shape.

This character was assessed by determining the curvature of a point’s basal edge. Curvature was measured using Collins’^46^ Index of Curvature, which divides the value *a*, the straight-line distance between the two basal ears of a point, into the value *b*, the maximum perpendicular distance between that line and the interior surface of the point’s basal edge (Fig. 5). The larger the value, the greater the curvature. For this character, we recognized three character states:

1. Flat (0.000–0.162)
2. Concave (0.163–0.323)
3. Very concave (0.324–0.486)

Character III. Basal-Indentation Ratio.

This character is the ratio between the medial length of a point and its maximum length (Fig. 5). The smaller the ratio, the smaller the basal indentation. For this character, we recognized three character states:

1. Shallow (0.0000–0.0952)
2. Deep (0.0953–0.1904)
3. Very deep (0.1905–0.2856)

Character IV. Constriction Ratio.

This character is the ratio between the minimum blade width (proximal to the point of maximum blade width) and the maximum blade width (Fig. 5). The larger the ratio, the greater the degree of constriction. For this character, we recognized three character states:

1. Little or no waist (0.000–0.131)
2. Waist (0.132–0.262)
3. Prominent waist (0.263–0.394)

Character V. Outer Tang (Basal Ear) Angle.

This character was assessed by measuring the degree of tang (basal ear) expansion relative to the long axis of a point (Fig. 5). For this character, we recognized four character states:

1. Diverging (both tangs larger than 92 degrees)
2. Parallel (both tangs between 88 and 92 degrees)
3. Converging (both tangs smaller than 88 degrees)
4. Multistate

Character VI. Tang (Basal Ear) Tip Shape.

This character was assessed by determining whether the tangs (basal ears) of a point were pointed or not. “Pointed” was defined using Eren *et al*.’s^47^ “spur procedure.” A tang (basal ear) was determined to be “pointed” if it extended through a box 3 mm by 1 mm (Fig. 5). This procedure yielded three character states:

1. Both pointed
2. Both round
3. Multistate

Character VII. Length/Width Ratio.

This character was assessed by dividing a point’s maximum length by its maximum width (Fig. 5). For this character, we recognized three character states:

1. Broad (0.000–2.437)
2. Narrow (2.438–3.354)
3. Very narrow (3.355–4.795)

The paradigmatic classification specified above produced 1944 possible classes, of which 133 were represented in our sample of 292 EP points (Supplementary Table 1).

### Diversity Assessment and Analysis

#### The three most commonly used diversity measures

We consider in our analysis three diversity measures that have been widely used in many disciplines. The three measures are in units of “class equivalents” or “effective number of classes” and possess the same intuitive mathematical properties as species richness. These three measures, as briefly described below, are instances of a single class of diversity measures, differing only by an integer exponent, *q*, called the *diversity order*; see Gotelli and Chao^48^, Chao *et al*.^49^, and Eren *et al*.^42^ for details.

1. *Class richness* (or diversity of order *q* = 0). the number of classes represented by the individuals (specimens in our case) in an assemblage. Not all classes are likely to be detected in a sample from the assemblage, so class richness includes not only the detected classes, but also classes present in the assemblage but not detected in the sample. This measure treats classes equally. When many rare classes remain undetected in the data, the number of detected classes in the sample severely underestimates the true species richness. In this case, class richness is statistically difficult to estimate. The best we can do is to infer a minimum level of class richness. We adopt the Chao1 estimator^50^ as the estimated asymptote of class richness. The Chao1 estimator is a non-parametric lower bound in the sense that it is universally valid for all types of class-abundance distributions. It generally works satisfactorily as a point estimator if the sample size is sufficiently large or the rare classes are approximately homogeneous in detection probabilities.
2. *Shannon diversity* (or common class richness, or diversity of order *q* = 1). Mathematically, Shannon diversity is expressed as the exponential of Shannon entropy. This transformation converts Shannon entropy (in units of information) to “class equivalents.” Shannon diversity treats individuals equally and thus it counts classes in proportion to their relative abundances; it can be interpreted as the effective number of common classes. Here “effective” means the number of equally abundant classes that would be needed to produce the given Shannon entropy of the actual assemblage^51^,^52^. Because the undetected classes in a sample are usually those with low relative abundances, and thus the effect of undetected species is generally limited, Shannon diversity can be statistically estimated with low bias. We adopt the low-bias non-parametric estimator proposed by Chao *et al*.^53^ as the estimated asymptote of Shannon diversity.
3. *Simpson diversity* (or dominant class richness, or diversity of order *q* = 2). Simpson diversity is expressed as the inverse of the Simpson repeat rate (i.e., the probability that two randomly selected individuals belong to the same class). This transformation converts the Gini-Simpson index (which is a probability) to “class equivalents”^51^,^52^. This measure disproportionately discounts rare classes and emphasizes the very common or dominant ones. It can thus be interpreted as the effective number of dominant or very common classes. Since dominant classes always appear in samples and undetected classes are discounted, Simpson diversity can often be quite accurately measured. In our analyses, we use the nearly unbiased estimator^46^ as the estimated asymptote of Simpson diversity.

#### Making fair comparisons of diversities between the East and the West

Based on Table 1a of the main text, the sample size in the East is lower than that in the West (138 vs. 154); the sample completeness (as measured by sample coverage) in the East is also lower (56.7% vs. 67.7%). Here sample coverage (or simply *coverage*) is an objective measure of sample completeness originally developed by the founder of modern computer science, Alan Turing, and his colleague I. J. Good^54^,^55^,^56^ (see ref. 41 for details). The estimated coverage values in Table 1a were obtained by an estimator^57^ that is more accurate than Turing’s original estimator. Table 1b and c show, for each diversity measure, that the observed diversity value in the East is higher than the West. However, it is well known that observed diversity, especially class richness, depends on sample size and sample completeness, so they cannot be fairly compared; standardization is needed. Two types of standardization allow fair comparisons of diversities among multiple assemblages, reducing the bias that arises from unequal sampling effort and incomplete samples that miss many species^49^.

#### Sample-size-based rarefaction, and extrapolation up to a maximum size

For each diversity measure, we standardized by estimating diversity for a fixed sample size, which can be smaller than an observed sample (traditional rarefaction) or larger than an observed sample (extrapolation). Then we constructed, for each sample, a seamless rarefaction and extrapolation sampling curve as a function of sample size. For species richness, the size can be extrapolated, at most, to double or triple the minimum observed sample size. For Shannon diversity and Simpson diversity, if data are not too sparse, the extrapolation of each measure can be reliably extended to infinity to attain the previously mentioned estimated asymptote.

#### Coverage-based rarefaction and extrapolation up to a maximum coverage

In this approach, we standardized samples by matching their sample completeness (as measured by sample coverage). Chao and Jost^58^ derived an analytic method for estimating sample coverage for rarified samples and extrapolated samples. The seamless rarefaction and extrapolation curve plots the diversity estimates as a function of sample coverage up to a maximum coverage. For species richness, the maximum coverage was selected as the coverage of the maximum size used in the sample-size-based sampling curve. For Shannon diversity and Simpson diversity, if data are not sparse, the extrapolation can often be extended to the coverage of unity to attain the previously mentioned estimated asymptote.

In both sample-size- and coverage-based sampling curves, extrapolation was guided by the previously mentioned estimated asymptote of each diversity measure. Chao *et al*.^49^ introduced a bootstrap method to construct 95% confidence intervals associated with each estimated diversity measure. Generally, for any fixed sample size or any degree of completeness in the comparison, if two 95% confidence intervals do not overlap, then significant differences between the expected diversities (whether interpolated or extrapolated) are guaranteed. However, overlapping intervals do not guarantee non-significance^57^; in this case, the data are inconclusive. All diversity estimates and confidence intervals, along with the plots of rarefaction/extrapolation sampling curves, can be obtained by using the software iNEXT^59^ which is available in CRAN and also from Anne Chao’s website.

In the left panels of Fig. 3, we compare the East and the West for each diversity measure at equivalent sample sizes by means of sample-size-based rarefaction and extrapolation sampling curves. In the right panels of Fig. 3, we compare the East and the West for each diversity measure at equivalent sample coverage by means of coverage-based sampling curves. Both types of sampling curve clearly show that the diversity curve for the East consistently lies above the curve of the West for all three measures, revealing that that, for any standardized sample size or coverage, the diversity of the East is higher than the West. Whether the difference is statistically significant depends on the measure and on coverage, as described below.

For Shannon diversity (middle row of Fig. 3) and Simpson diversity (lower row of Fig. 3), the 95% confidence intervals for the two areas do not overlap regardless of the type of rarefaction/extrapolation sampling curves. This non-overlap implies that our data provide sufficient evidence that the East is significantly more diverse than the West for common class richness and also for dominant class richness. Since the extrapolation for these two diversity measures can usually be reliably extended to attain the asymptote^49^, our conclusion is valid not only for the range of samples sizes and of sample coverage values considered in Fig. 3 but also for any sample size and any range of coverage up to complete coverage. This conclusion for the asymptotes is supported by examining the confidence intervals for the estimated asymptotes of diversities: Table 1b and c show that the estimated asymptote of Shannon diversity in the East was 158.9 with a 95% confidence interval of (119.9, 197.8), whereas the corresponding asymptote and confidence interval for the West were 81.5 and (55.0, 107.9). Because the two intervals do not overlap, we conclude that the East is significantly more diverse in common classes, even for the entire assemblage (i.e., as sample size tends to infinity and coverage tends to unity). Similar disjoint confidence intervals are also shown for Simpson diversity, implying that the East is also significantly more diverse in dominant classes for the entire assemblage.

For class richness, the confidence intervals of the two areas overlap for sample-size-based sampling curve for size >200 and are thus inconclusive (upper left row of Fig. 3). By contrast, the two coverage-based intervals are disjoint (upper right row), and thus provide sufficient evidence that the East is significantly more diverse than the West for the range of sample coverage up to 80% (i.e., up to 80% fraction of the assemblages), but significance cannot be demonstrated for larger assemblages due to undetected classes, even though overall the East is richer than the West in this measure as well. As shown in Table 1b and c of (main text), the confidence intervals of the two asymptotes do overlap.

In summary, the data with the definition of character states provide sufficient evidence that the East is significantly more diverse than the West for (effective) common and dominant classes of projectile points for any standardized samples size and sample coverage up to asymptotes. For class richness, the data support the same significant difference up to 80% fraction of the assemblages, but not for larger fractions, due to undetected classes.

### Heterogeneity Measure: Coefficient of Variation (CV)

In statistics, the degree or extent of heterogeneity in class abundances can be quantified by the coefficient of variation (CV), which is the ratio of the standard deviation, among class abundances, to the mean of class abundances. This measure takes values between 0 and infinity. When all class abundances are equal (the homogeneous case), CV = 0 because the standard deviation is 0. A larger value of CV indicates a higher degree of heterogeneity among class abundances. The mathematical formula for the CV measure in our application can be expressed as follows. Suppose there are *S* classes indexed by 1, 2, …, *S*, and the number of specimens (abundance) in the *i-*th class is *X*_*i*_. Then the CV can be expressed as


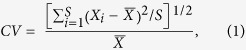


where 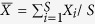 denotes the mean of the class abundance set {*X*_1_, *X*_2_, …, *X*_*S*_ }. The numerator in the above formula is the standard deviation of the class abundance set.

In practical applications, since class richness *S* and class abundances are unknown, statistical estimation is needed to infer the CV value. In Table 1 we give the estimated CV values for the East and the West, computed using the R package SpadeR (Species-Richness Prediction And Diversity Estimation in R), which is available from CRAN^56^. An R-based interactive online version SpadeR Online is available from Anne Chao’s website. The estimated CV for the East is 0.632, whereas the estimated CV for the West is 1.387, signifying that class abundance in the West is uneven, with some point designs being very abundant while other designs are rare, whereas the class abundance in the East is relatively more even. See Chao and Lee^60^ for the estimation method.

### Network Analysis

We used the point classes represented in our sample of 49 EP assemblages to construct networks. Assemblages were designated as nodes in our analyses, with shared point classes between nodes connected by *ties* (links). The EP point-class networks are binary, with symmetric, undirected ties among nodes containing similar classes. Ties were weighted using the Sørensen Index, which normalizes the richness of point classes among two assemblages^61^. To calculate the Sørensen Index we divided the number of shared point classes between two assemblages by the average class richness of the two assemblages. We constructed network graphs using the layout procedure in NetDraw version 2.089^62^. We employed the spring embedding method using geodesic distances and 100 iterations, using node repulsion and equal edge length as the layout criteria to visualize the networks.

We ran analyses on the overall point-class network using Ucinet version 6.232^63^. First, we calculated the average density of the overall point-class network. The average density of a network is the proportion of all possible ties present in a network. Networks with greater densities are better connected, and social and biological information flows more readily within dense networks^64^. The results of our analyses demonstrate that the point-class network has a density of 0.0331 (±0.086SD; average bootstrap density based on 5000 samples = 0.0524). Second, we calculated the presence of components and isolates within the overall lithic network. Components have multiple nodes with shared point classes, and isolates are nodes that have points from only a single class that does not occur in any other assemblage. The results of the component analysis of the overall dataset of 49 nodes revealed four distinct components, three of which are isolated nodes, whereas the remaining network contains 46 nodes. The isolates are the points from the Escapule and Leikum sites in southern Arizona, and the Whipple assemblage from New Hampshire.

## Additional Information

**How to cite this article** Buchanan, B. et al. Environment-induced changes in selective constraints on social learning during the peopling of the Americas. *Sci. Rep.* **7**, 44431; doi: 10.1038/srep44431 (2017).

**Publisher's note:** Springer Nature remains neutral with regard to jurisdictional claims in published maps and institutional affiliations.

## Supplementary Material

Supplementary Information

Supplementary Dataset 1

## Figures and Tables

**Figure 1:**
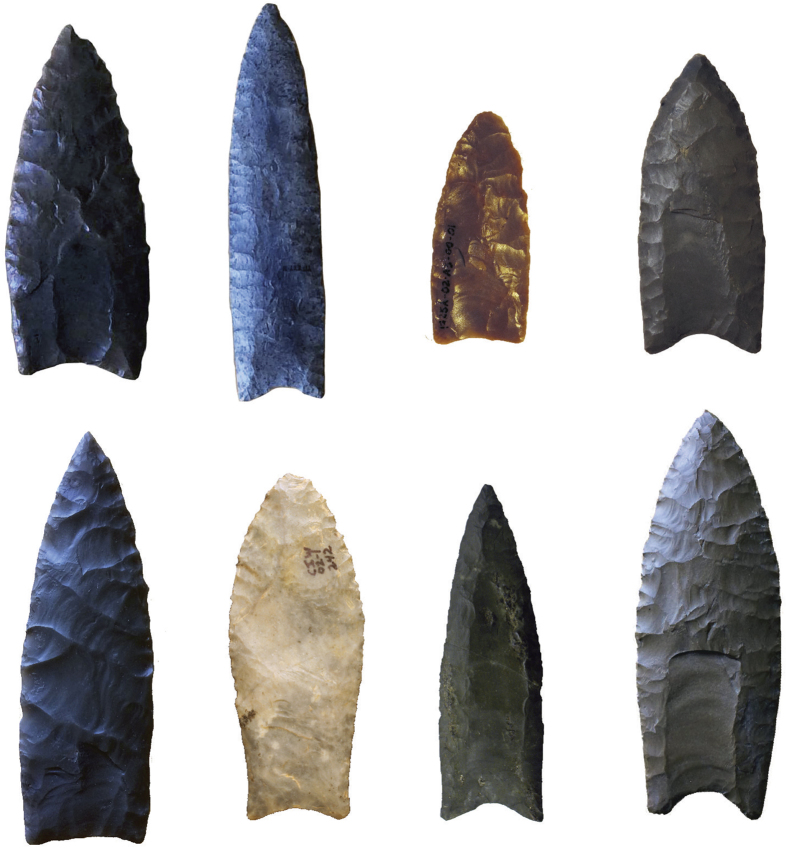
Clovis points from various North American sites. Top row, left to right: Dent, Colorado; Kimmswick, Missouri; Paleo Crossing, Ohio; Welling, Ohio. Bottom row, left to right: Blackwater Draw, New Mexico; Carson-Conn-Short, Tennessee (photo courtesy of Ashley Smallwood [Smallwood, A. M. Clovis technology and settlement in the American Southeast: using biface analysis to evaluate dispersal models. *Am. Antiquity* **77**, 689–713 (2012)]); Bull Brook, Massachusetts; Vail, Maine.

**Table 1:** Data summary for the East and West, with statistical inference for estimated asymptotes of diversities.

(a) Data summary for the East and West.																	
(*f*_*k*_ denotes the number of classes represented by exactly *k* individuals in the sample)																	
Area	Sample size *n*	Observed class richness	Sample complete-ness	CV	*f*_1_	*f*_2_	*f*_3_	*f*_4_	*f*_5_	*f*_6_	*f*_7_	*f*_8_	*f*_9_	*f*_10_	*f*_11_	*f*_12_	*f*_13_
East	138	88	56.7%	0.632	60	15	7	4	1	1	0	0	0	0	0	0	0
West	154	71	68.9%	1.387	48	8	7	2	0	0	1	1	0	1	1	1	1
**(b) Observed diversities and estimated asymptotic diversities in the East.**																	
			**Observed diversity**			**Estimated asymptote**			**Estimated s.e.**			**95% lower confidence interval**			**95% upper confidence interval**		
Class richness			88.0			207.1			44.8			146.4			331.2		
Shannon diversity (common class richness)			74.6			158.9			19.9			119.9*			197.8*		
Simpson diversity (dominant class richness)			61.8			111.2			14.8			82.2*			140.2*		
**(c) Observed diversities and estimated asymptotic diversities in the West.**																	
Class richness			71.0			214.1			66.4			131.2			411.0		
Shannon diversity (common class richness)			45.4			84.0			12.9			58.7*			109.2*		
Simpson diversity (dominant class richness)			28.9			35.3			5.3			28.9*			45.8*		

^*^Interval does not overlap with the interval for the West.

^*^Interval does not overlap with the interval for the East.
