# Supplementary material for: Environment-induced changes in selective constraints on social learning during the peopling of the Americas
Source: Sci Rep. 2017 Mar 16;7:44431. doi: 10.1038/srep44431 (PMC5353571; doi:10.1038/srep44431)
Supplement: Supplementary Information [file srep44431-s1.pdf]

Supplementary Materials for

Environment-induced erosion of social learning during the peopling of the Americas

Briggs Buchanan[1], Anne Chao[2], Chun-Huo Chiu[2], Robert K. Colwell[3,4], Michael J. O'Brien[5], Angelia Werner[6], Metin I. Eren[6,7]

1. Department of Anthropology, University of Tulsa, Tulsa, OK, 74104, U.S.A.
2. Institute of Statistics, National Tsing Hua University, Hsin-Chu, 30043, Taiwan
3. Department of Ecology and Evolutionary Biology, University of Connecticut, Storrs, CT, 06269-3043, U.S.A.
4. Museum of Natural History, University of Colorado, Boulder, CO, 80309 USA
5. Department of Anthropology, University of Missouri, Columbia, MO, 65211, U.S.A.
6. Department of Anthropology, Kent State University, Kent, OH, 44242, U.S.A.
7. Department of Archaeology, Cleveland Museum of Natural History, Cleveland, OH, 44106, U.S.A.

Correspondence to: briggs-buchanan@utulsa.edu, meren@kent.edu

This PDF file includes:

Figures S1 to S14
Tables S1 to S15

Class richness (q = 0)

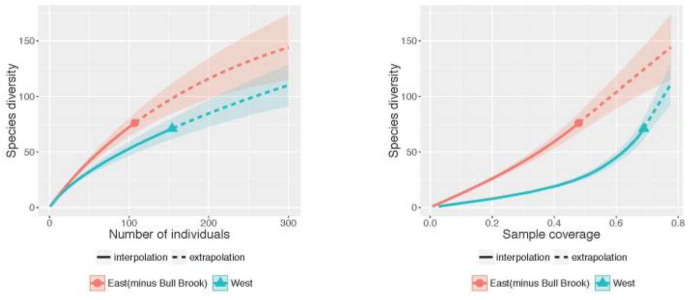

Shannon diversity (q = 1)

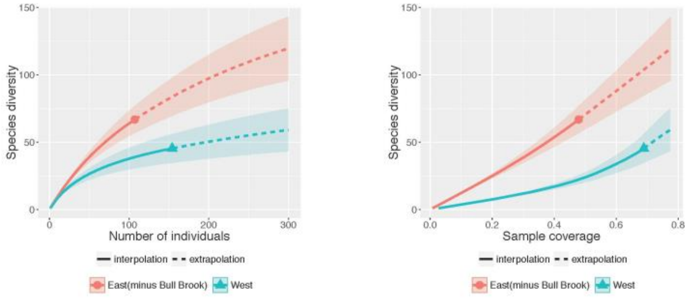

Simpson diversity (q = 2)

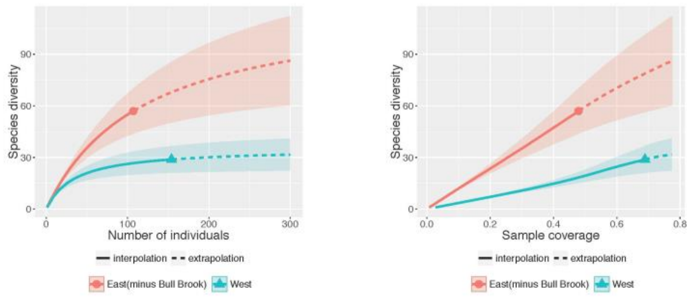

Figure S1 (Minus Bull Brook). Comparison of sample-size-based (left panels) and sample-coverage-based (right panels) rarefaction and extrapolation for class richness (upper panels), Shannon diversity (middle panels) and Simpson diversity (lower panels) for the East and the West data. See the caption for Fig. 3 of the main text for complete explanation of the graphs and symbols.

Class richness (q = 0)

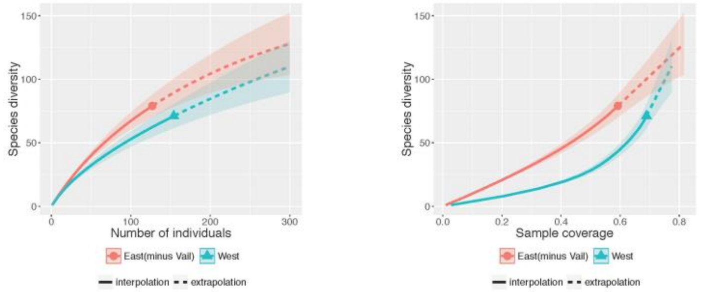

Shannon diversity (q = 1)

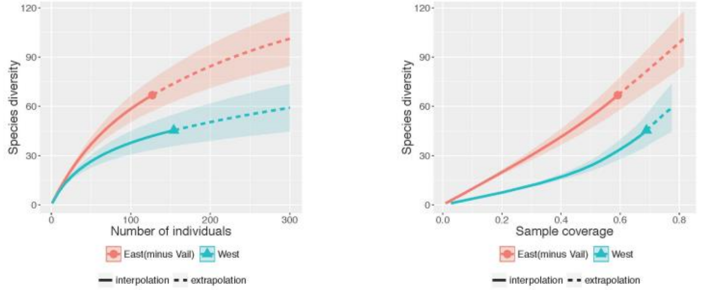

Simpson diversity (q = 2)

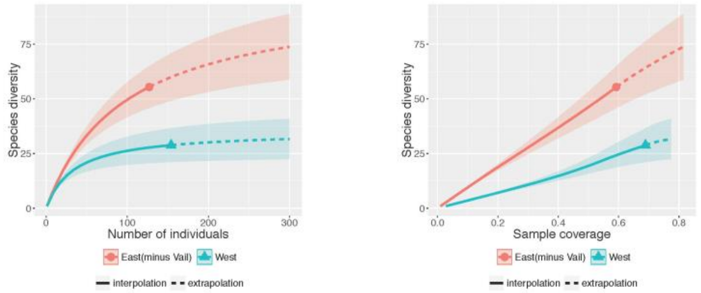

Figure S2 (Minus Vail). Comparison of sample-size-based (left panels) and sample-coverage-based (right panels) rarefaction and extrapolation for class richness (upper panels), Shannon diversity (middle panels), and Simpson diversity (lower panels) for the East and the West data. See Fig. 3 of the main text for complete explanation of the graphs and symbols.

Class richness (q = 0)

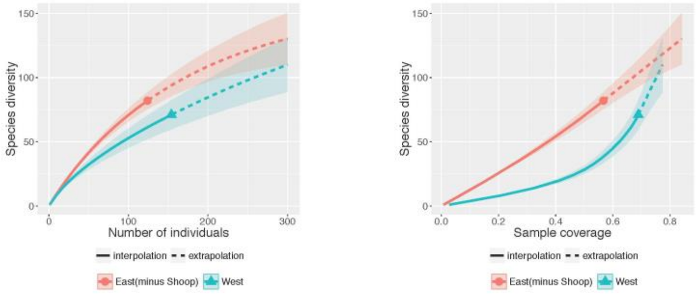

Shannon diversity (q = 1)

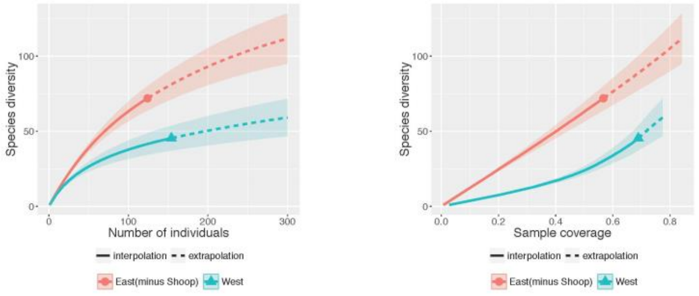

Simpson diversity (q = 2)

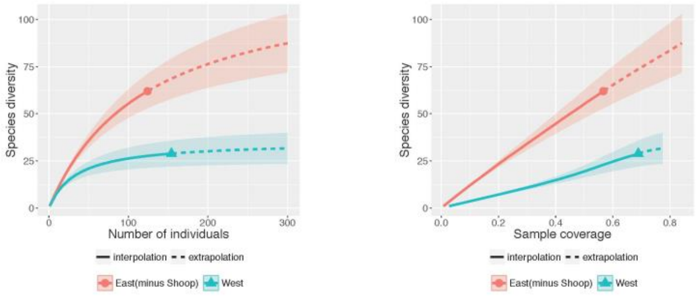

Figure S3 (Minus Shoop). Comparison of sample-size-based (left panels) and sample-coverage-based (right panels) rarefaction and extrapolation for class richness (upper panels), Shannon diversity (middle panels), and Simpson diversity (lower panels) for the East and the West data. See Fig. 3 of the main text for complete explanation of the graphs and symbols.

Class richness (q = 0)

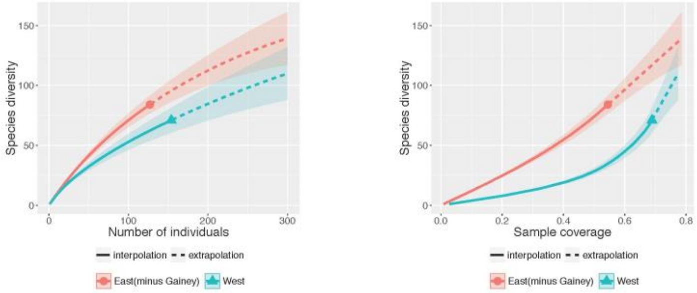

Shannon diversity (q = 1)

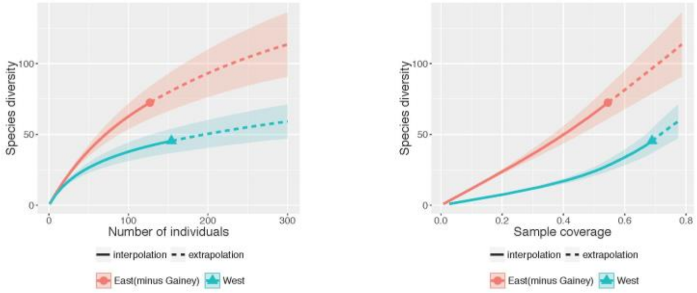

Simpson diversity (q = 2)

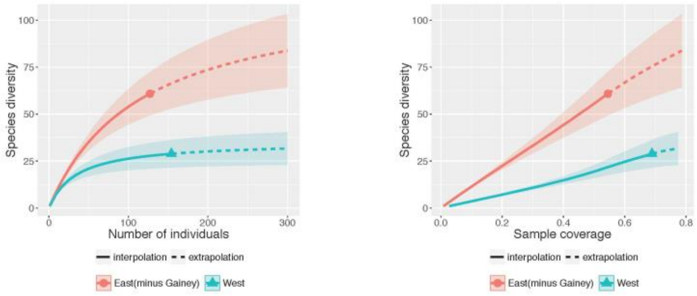

Figure S4 (Minus Gainey). Comparison of sample-size-based (left panels) and sample-coverage-based (right panels) rarefaction and extrapolation for class richness (upper panels), Shannon diversity (middle panels), and Simpson diversity (lower panels) for the East and the West data. See Fig. 3 of the main text for complete explanation of the graphs and symbols.

Class richness (q = 0)

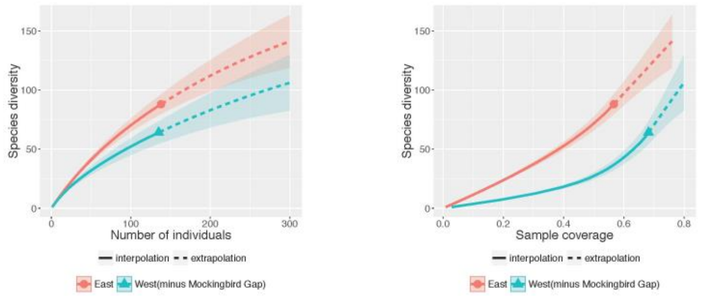

Shannon diversity (q = 1)

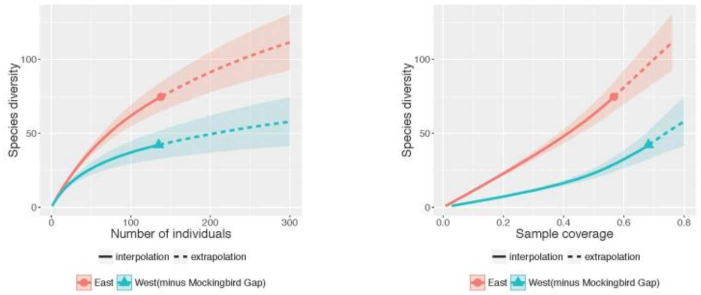

Simpson diversity (q = 2)

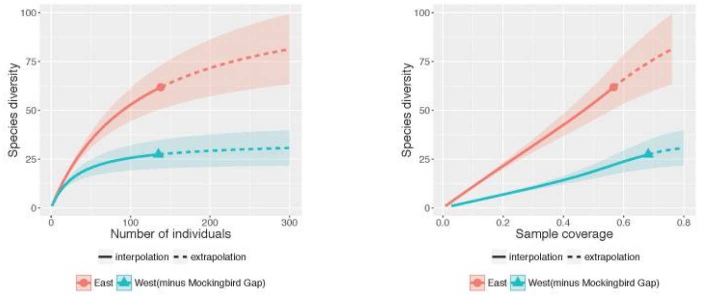

Figure S5 (Minus Mockingbird Gap). Comparison of sample-size-based (left panels) and sample-coverage-based (right panels) rarefaction and extrapolation for class richness (upper panels), Shannon diversity (middle panels), and Simpson diversity (lower panels) for the East and the West data. See Fig. 3 of the main text for complete explanation of the graphs and symbols.

Class richness (q = 0)

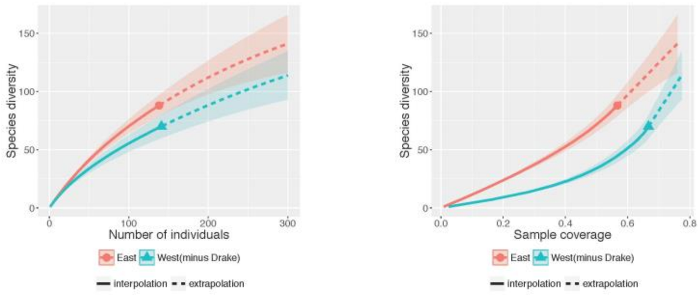

Shannon diversity (q = 1)

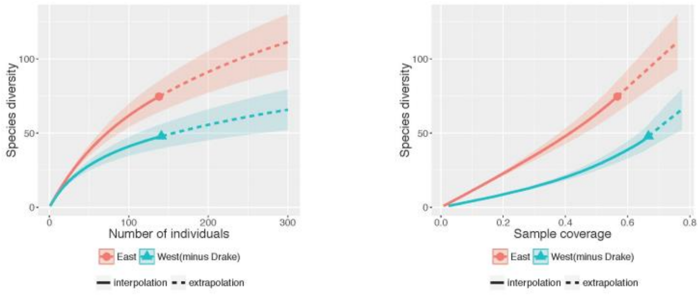

Simpson diversity (q = 2)

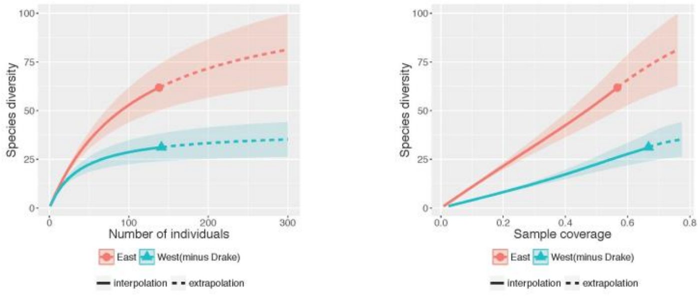

Figure S6 (Minus Drake). Comparison of sample-size-based (left panels) and sample-coverage-based (right panels) rarefaction and extrapolation for class richness (upper panels), Shannon diversity (middle panels), and Simpson diversity (lower panels) for the East and the West data. See Fig. 3 of the main text for complete explanation of the graphs and symbols.

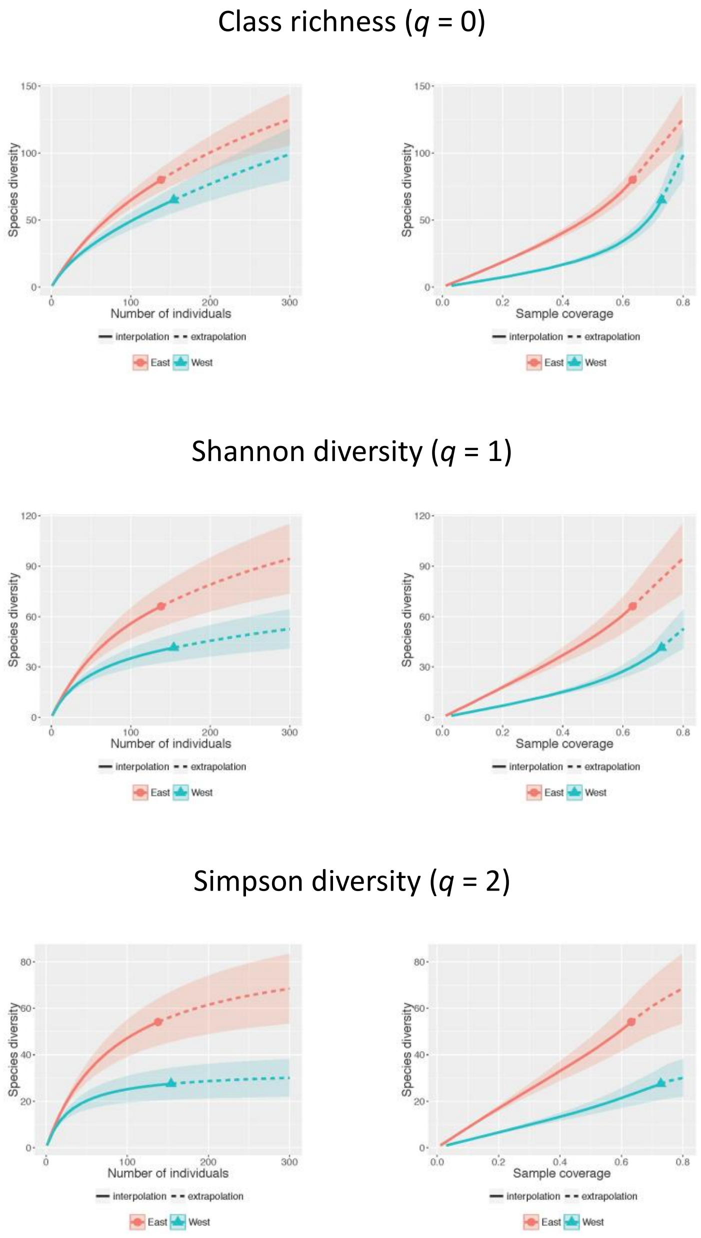

Figure S7 (Minus 1st character). Comparison of sample-size-based (left panels) and sample-coverage-based (right panels) rarefaction and extrapolation for class richness (upper panels), Shannon diversity (middle panels), and Simpson diversity (lower panels) for the East and the West data. See Fig. 3 of the main text for complete explanation of the graphs and symbols.

Class richness (q = 0)

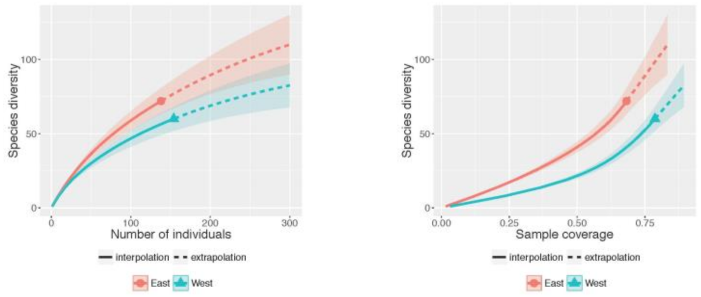

Shannon diversity (q = 1)

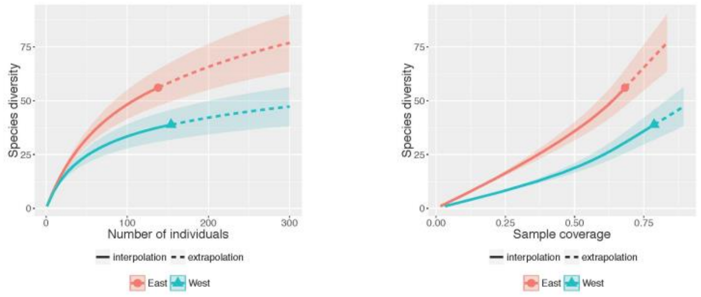

Simpson diversity (q = 2)

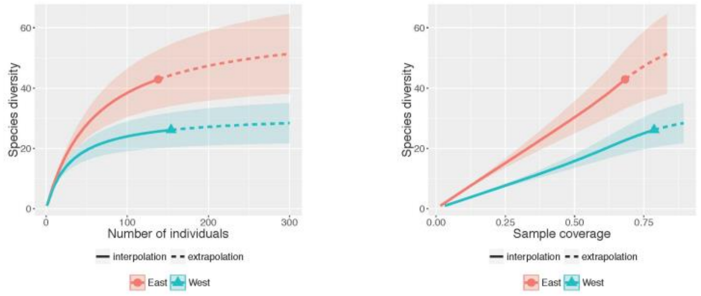

Figure S8 (Minus 2nd character). Comparison of sample-size-based (left panels) and sample-coverage-based (right panels) rarefaction and extrapolation for class richness (upper panels), Shannon diversity (middle panels), and Simpson diversity (lower panels) for the East and the West data. See Fig. 3 of the main text for complete explanation of the graphs and symbols.

Class richness (q = 0)

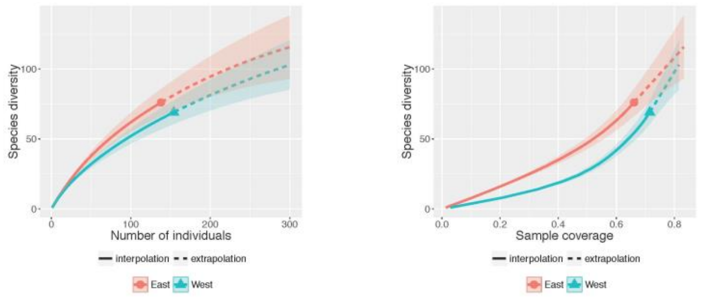

Shannon diversity (q = 1)

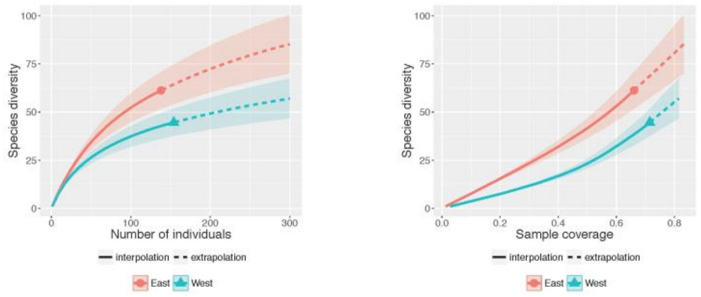

Simpson diversity (q = 2)

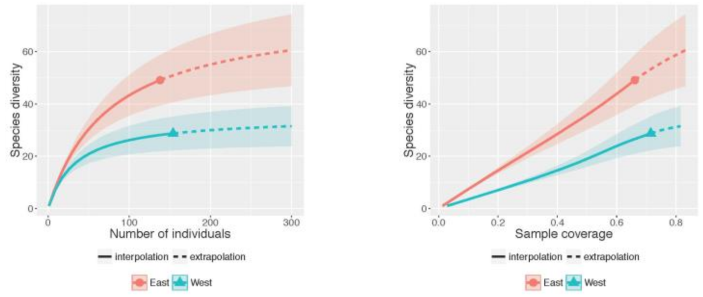

Figure S9 (Minus 3rd character). Comparison of sample-size-based (left panels) and sample-coverage-based (right panels) rarefaction and extrapolation for class richness (upper panels), Shannon diversity (middle panels), and Simpson diversity (lower panels) for the East and the West data. See Fig. 3 of the main text for complete explanation of the graphs and symbols.

Class richness (q = 0)

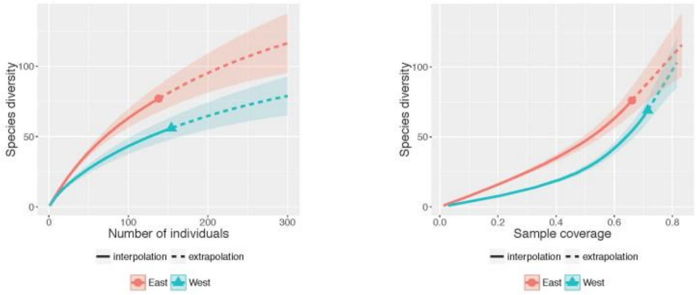

Shannon diversity (q = 1)

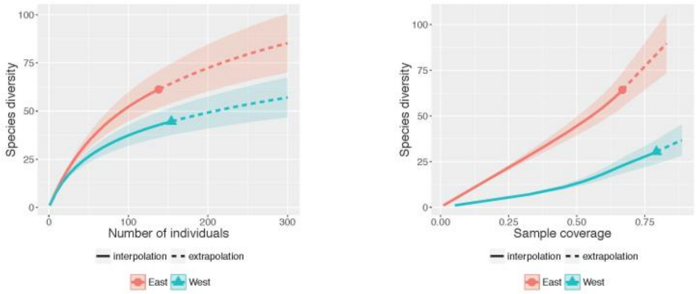

Simpson diversity (q = 2)

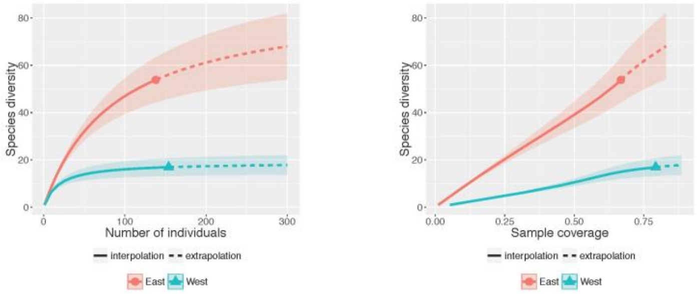

Figure S10 (Minus 4th character). Comparison of sample-size-based (left panels) and sample-coverage-based (right panels) rarefaction and extrapolation for class richness (upper panels), Shannon diversity (middle panels), and Simpson diversity (lower panels) for the East and the West data. See Fig. 3 of the main text for complete explanation of the graphs and symbols.

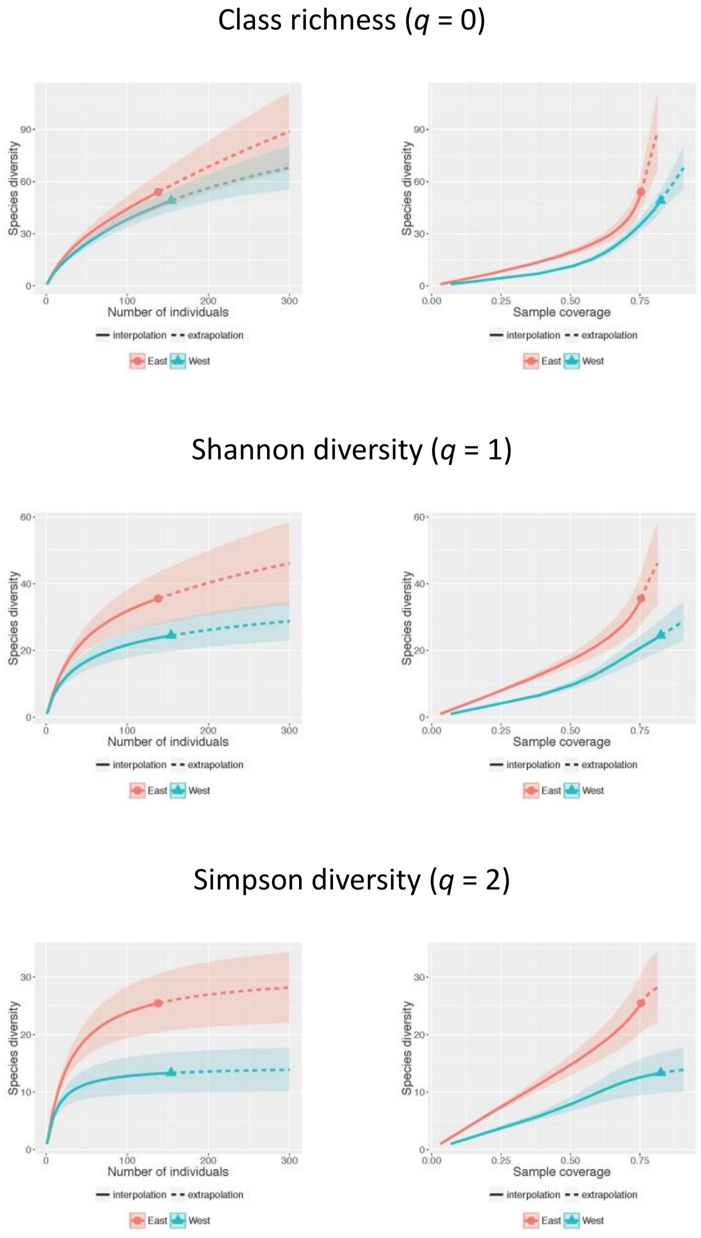

Figure S11 (Minus 5th character). Comparison of sample-size-based (left panels) and sample-coverage-based (right panels) rarefaction and extrapolation for class richness (upper panels), Shannon diversity (middle panels), and Simpson diversity (lower panels) for the East and the West data. See Fig. 3 of the main text for complete explanation of the graphs and symbols.

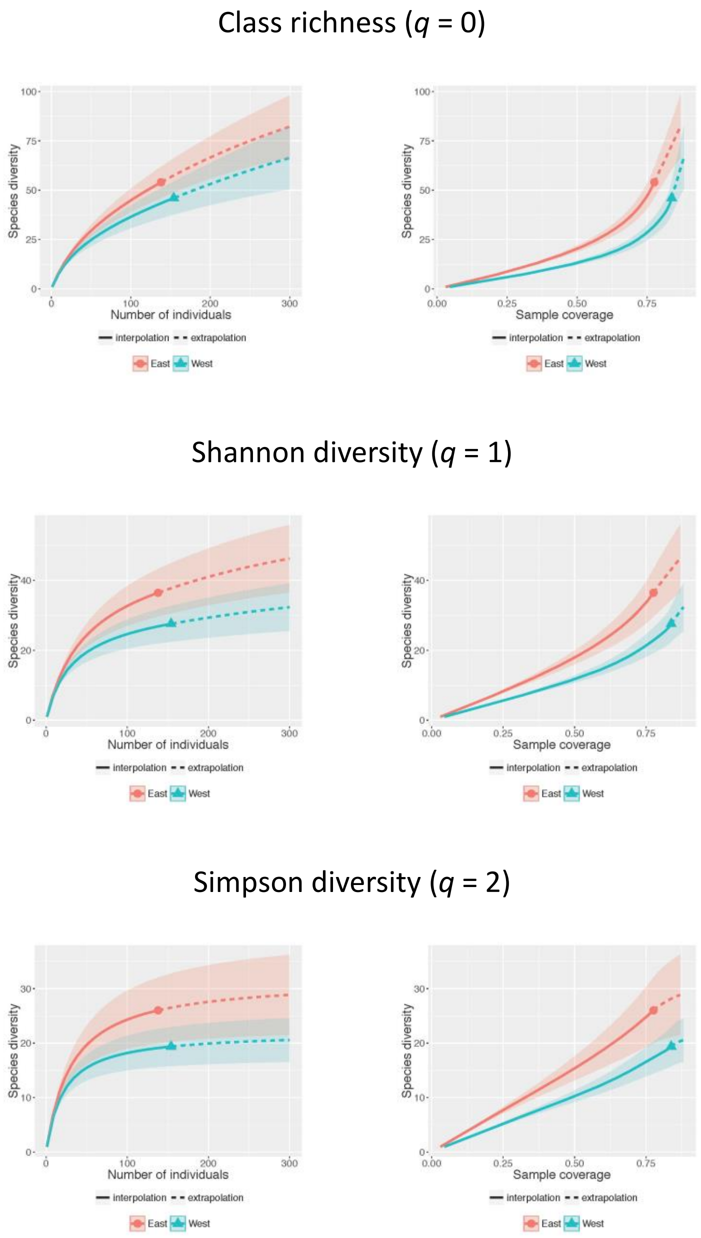

Figure S12 (Minus 6th character). Comparison of sample-size-based (left panels) and sample-coverage-based (right panels) rarefaction and extrapolation for class richness (upper panels), Shannon diversity (middle panels), and Simpson diversity (lower panels) for the East and the West data. See Fig. 3 of the main text for complete explanation of the graphs and symbols.

Class richness (q = 0)

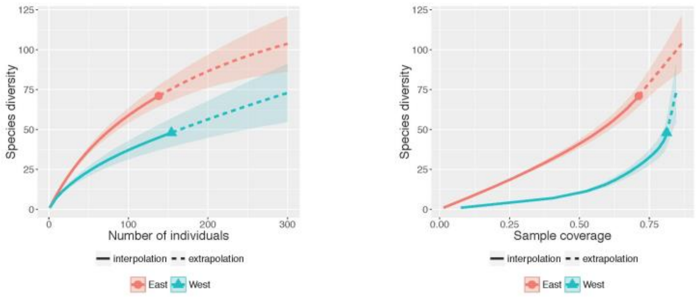

Shannon diversity (q = 1)

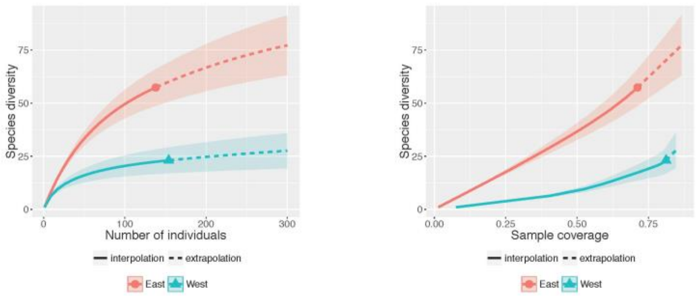

Simpson diversity (q = 2)

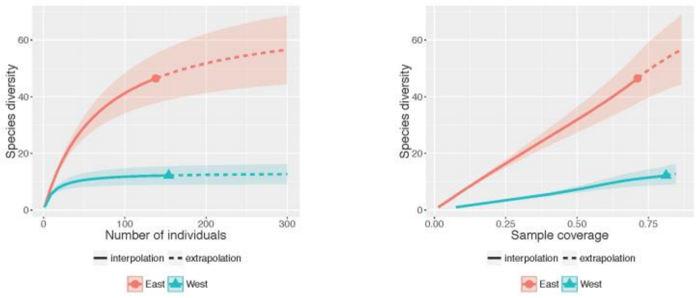

Figure S13 (Minus 7th character). Comparison of sample-size-based (left panels) and sample-coverage-based (right panels) rarefaction and extrapolation for class richness (upper panels), Shannon diversity (middle panels), and Simpson diversity (lower panels) for the East and the West data. See Fig. 3 of the main text for complete explanation of the graphs and symbols.

Class richness (q = 0)

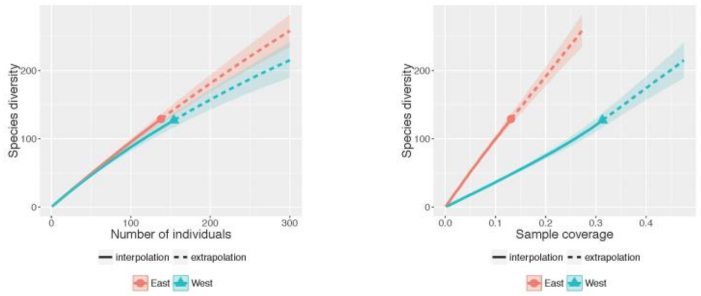

Shannon diversity (q = 1)

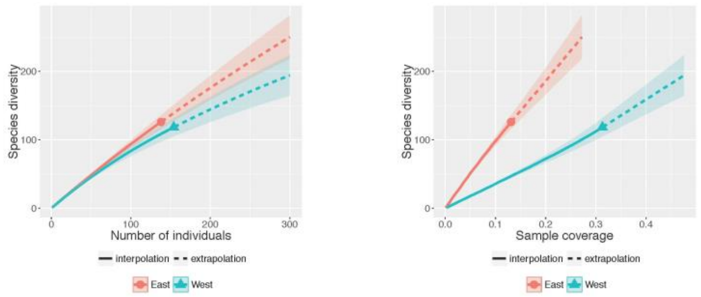

Simpson diversity (q = 2)

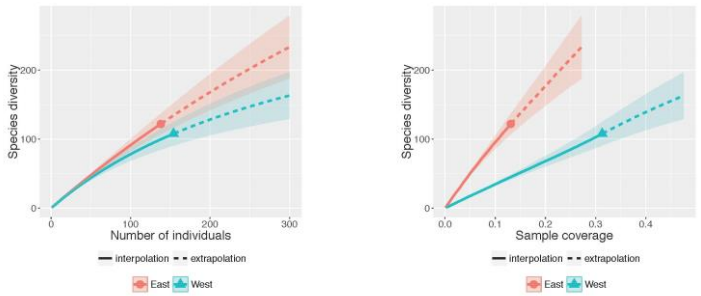

Figure S14 (Narrow definition of character states). Comparison of sample-size-based (left panels) and sample-coverage-based (right panels) rarefaction and extrapolation for class richness (upper panels), Shannon diversity (middle panels), and Simpson diversity (lower panels) for the East and the West data. See Fig. 3 of the main text for complete explanation of the graphs and symbols.

**Site**	**Region**	**# of points in analysis**	**Reference(s)**
**Adkins**	East	1	*S1*
**Anzick**	West	6	*S2-S4*
**Big Eddy**	West	2	*S5, S6*
**Blackwater Draw**	West	22	*S7-S12*
**Bull Brook**	East	31	*S13-S16*
**Bull Brook II**	East	2	*S17*
**Butler**	East	2	*S18*
**Cactus Hill**	East	5	*S19*
**Carson-Conn-Short**	East	2	*S20-S22*
**Colby**	West	3	*S23*
**Crook County**	West	1	*S24, S25*
**Debert**	East	5	*S26, S27*
**Dent**	West	2	*S28-S30*
**Domebo**	West	3	*S31*
**Drake**	West	13	*S32*
**East Wenatchee**	West	13	*S33, S34*
**El Fin del Mundo**	West	6	*S35*
**Escapule**	West	1	*S36*
**Fenn**	West	16	*S37, S38*
**Gainey**	East	11	*S18, S39, S40*
**Gault**	West	5	*S41-S43*
**Indian Creek**	West	1	*S44, S45*
**Jake Bluff**	West	4	*S46*
**Kimmswick**	West	3	*S47, S48*
**Kincaid**	West	1	*S49, S50*
**Lamb**	East	5	*S51*
**Lange–Ferguson**	West	2	*S52, S53*
**Lehner**	West	9	*S54*
**Leikum**	West	1	*S55, S56*
**Miami**	West	3	*S57-S59*
**Mockingbird Gap**	West	19	*S60-S62*
**Murray Springs**	West	5	*S63-S64*
**Naco**	West	7	*S65*
**Nobles Pond**	East	5	*S66-S68*
**Paleo Crossing**	East	5	*S69-S72*
**Pavo Real**	West	1	*S73-S74*
**Potts**	East	1	*S75, S76*
**Rummells–Maske**	East	10	*S77, S78*
**Shawnee–Minisink**	East	2	*S79-S81*
**Sheriden**	East	1	*S82-S84*
**Shoop**	East	14	*S84, S85*
**Simon**	West	5	*S86-S89*
**Sloth Hole**	East	2	*S90, S91*
**Udora**	East	2	*S92*
**Vail**	East	11	*S93-S95*

**Welling**	East	8	*S96*
**West Athens Hill**	East	5	*S97, S98*
**Whipple**	East	2	*S99-S101*
**Williamson**	East	7	*S102-S104*

Table S1. Clovis assemblages and number of complete points used in the analysis.

(a) Data summary of the East (minus Bull Brook) and the West (f_k denotes the number of classes represented by exactly *k* individuals in the sample).

Area	Sample size *n*	Observed class richness	Sample complete-ness	CV	f_1	f_2	f_3	f_4	f_5	f_6	f_7	f_8	f_9	f_{10}	f_{11}	f_{12}	f_{13}
East (minus Bull Brook)	107	76	4.79%	0.567	56	13	4	2	1	0	0	0	0	0	0	0	0
West	154	71	68.9%	1.387	48	8	7	2	0	0	1	1	0	1	1	1	1

(b) Observed diversities and the estimated asymptotes of diversities in the East (minus Bull Brook).

	Observed diversity	Estimated asymptote	Estimated s.e.	95% lower confidence interval	95% upper confidence interval
Class richness	76.0	195.5	47.3	132.6	328.4
Shannon diversity (common class richness)	66.8	164.4	28.0	109.6*****	219.2*****
Simpson diversity (dominant class richness)	57.0	120.7	19.2	83.1*****	158.3*****

***** Interval does not overlap with the interval for the West.

(c) Observed diversities and the estimated asymptotes of diversities in the West.

	Observed diversity	Estimated asymptote	Estimated s.e.	95% lower confidence interval	95% upper confidence interval
Class richness	71.0	214.1	66.4	131.2	411.0
Shannon diversity (common class richness)	45.4	84.0	12.9	58.7*	109.2*
Simpson diversity (dominant class richness)	28.9	35.3	5.3	28.9*	45.8*

***** Interval does not overlap with the interval for the East (minus Bull Brook).

Table S2 (Minus Bull Brook). Data summary for the East and the West, with statistical inference for estimated asymptotes of diversities.

(a) Data summary of the East (minus Vail) and the West (f_k denotes the number of classes represented by exactly k individuals in the sample).

Area	Sample size n	Observed class richness	Sample complete-ness	CV	f_1	f_2	f_3	f_4	f_5	f_6	f_7	f_8	f_9	f_{10}	f_{11}	f_{12}	f_{13}
East (minus Vail)	127	79	59.2%	0.609	52	15	6	4	1	1	0	0	0	0	0	0	0
West	154	71	68.9%	1.387	48	8	7	2	0	0	1	1	0	1	1	1	1

(b) Observed diversities and the estimated asymptotes of diversities in the East (minus Vail).

	Observed diversity	Estimated asymptote	Estimated s.e.	95% lower confidence interval	95% upper confidence interval
Class richness	79.0	168.4	35.2	121.5	267.1
Shannon diversity (common class richness)	66.8	133.5	15.1	103.9	163.0
Simpson diversity (dominant class richness)	55.4	97.6	14.6	69.0*	126.1*

* Interval does not overlap with the interval for the West.

(c) Observed diversities and the estimated asymptotes of diversities in the West.

	Observed diversity	Estimated asymptote	Estimated s.e.	95% lower confidence interval	95% upper confidence interval
Class richness	71.0	214.1	66.4	131.2	411.0
Shannon diversity (common class richness)	45.4	84.0	12.9	58.7	109.2
Simpson diversity (dominant class richness)	28.9	35.3	5.3	28.9*	45.8*

***** Interval does not overlap with the interval for the East (minus Vail).

Table S3 (Minus Vail). Data summary for the East and the West, with statistical inference for estimated asymptotes of diversities.

(a) Data summary of the East (minus Shoop) and the West (f_k denotes the number of classes represented by exactly k individuals in the sample).

Area	Sample size n	Observed class richness	Sample complete-ness	CV	f_1	f_2	f_3	f_4	f_5	f_6	f_7	f_8	f_9	f_{10}	f_{11}	f_{12}	f_{13}
East (minus Shoop)	124	82	56.7%	0.425	54	19	5	3	1	0	0	0	0	0	0	0	0
West	154	71	68.9%	1.387	48	8	7	2	0	0	1	1	0	1	1	1	1

(b) Observed diversities and the estimated asymptotes of diversities in the East (minus Shoop).

	Observed diversity	Estimated asymptote	Estimated s.e.	95% lower confidence interval	95% upper confidence interval
Class richness	82.0	158.1	28.5	119.5	236.7
Shannon diversity (common class richness)	71.9	144.0	16.6	111.4*	176.5*
Simpson diversity (dominant class richness)	62.0	123.0	16.5	90.7*	155.3*

* Interval does not overlap with the interval for the West.

(c) Observed diversities and the estimated asymptotes of diversities in the West.

	Observed diversity	Estimated asymptote	Estimated s.e.	95% lower confidence interval	95% upper confidence interval
Class richness	71.0	214.1	66.4	131.2	411.0
Shannon diversity (common class richness)	45.4	84.0	12.9	58.7*	109.2*
Simpson diversity (dominant class richness)	28.9	35.3	5.3	28.9*	45.8*

***** Interval does not overlap with the interval for the East (minus Shoop).

Table S4 (Minus Shoop). Data summary for the East and the West, with statistical inference for estimated asymptotes of diversities.

(a) Data summary of the East (minus Gainey) and the West (f_k denotes the number of classes represented by exactly k individuals in the sample).

Area	Sample size n	Observed class richness	Sample complete-ness	CV	f_1	f_2	f_3	f_4	f_5	f_6	f_7	f_8	f_9	f_{10}	f_{11}	f_{12}	f_{13}
East (minus Gainey)	127	84	54.5%	0.577	58	16	5	3	2	0	0	0	0	0	0	0	0
West	154	71	68.9%	1.387	48	8	7	2	0	0	1	1	0	1	1	1	1

(b) Observed diversities and the estimated asymptotes of diversities in the East (minus Gainey).

	Observed diversity	Estimated asymptote	Estimated s.e.	95% lower confidence interval	95% upper confidence interval
Class richness	84.0	188.3	39.2	135.2	296.6
Shannon diversity (common class richness)	72.4	155.6	20.2	116.0*	195.3*
Simpson diversity (dominant class richness)	60.9	116.0	17.7	81.4*	150.5*

* Interval does not overlap with the interval for the West.

(c) Observed diversities and the estimated asymptotes of diversities in the West.

	Observed diversity	Estimated asymptote	Estimated s.e.	95% lower confidence interval	95% upper confidence interval
Class richness	71.0	214.1	66.4	131.2	411.0
Shannon diversity (common class richness)	45.4	84.0	12.9	58.7*	109.2*
Simpson diversity (dominant class richness)	28.9	35.3	5.3	28.9*	45.8*

***** Interval does not overlap with the interval for the East (minus Gainey).

Table S5 (Minus Gainey). Data summary for the East and the West, with statistical inference for estimated asymptotes of diversities.

(a) Data summary of the East and the West (minus Mockingbird Gap) (f_k denotes the number of classes represented by exactly k individuals in the sample).

Area	Sample size n	Observed class richness	Sample complete-ness	CV	f_1	f_2	f_3	f_4	f_5	f_6	f_7	f_8	f_9	f_{10}	f_{11}	f_{12}
East	138	88	56.7%	0.632	60	15	7	4	1	1	0	0	0	0	0	0
West (minus Mockingbird Gap)	135	64	68.2%	1.323	43	8	7	0	0	0	2	1	0	1	1	1

(b) Observed diversities and the estimated asymptotes of diversities in the East.

	Observed diversity	Estimated asymptote	Estimated s.e.	95% lower confidence interval	95% upper confidence interval
Class richness	88.0	207.1	44.8	146.4	331.2
Shannon diversity (common class richness)	74.6	158.9	18.5	122.6*****	195.1*****
Simpson diversity (dominant class richness)	61.8	111.2	16.5	78.9*****	143.6*****

***** Interval does not overlap with the interval for the West (minus Mockingbird Gap).

(c) Observed diversities and the estimated asymptotes of diversities in the West (minus Mockingbird Gap).

	Observed diversity	Estimated asymptote	Estimated s.e.	95% lower confidence interval	95% upper confidence interval
Class richness	64.0	178.7	54.6	111.3	342.2
Shannon diversity (common class richness)	42.1	77.4	11.9	54.0*	100.8*
Simpson diversity (dominant class richness)	27.4	34.1	5.9	27.4*	45.6*

***** Interval does not overlap with the interval for the East.

Table S6 (Minus Mockingbird Gap). Data summary for the East and the West, with statistical inference for estimated asymptotes of diversities.

(a) Data summary of the East and the West (minus Drake) (f_k denotes the number of classes represented by exactly k individuals in the sample).

Area	Sample size n	Observed class richness	Sample complete-ness	CV	f_1	f_2	f_3	f_4	f_5	f_6	f_7	f_8	f_9	f_{10}	f_{11}	f_{12}	f_{13}
East	138	88	56.7%	0.632	60	15	7	4	1	1	0	0	0	0	0	0	0
West (minus Drake)	141	70	66.8%	1.280	47	8	8	2	0	1	1	0	0	2	0	0	1

(b) Observed diversities and the estimated asymptotes of diversities in the East.

	Observed diversity	Estimated asymptote	Estimated s.e.	95% lower confidence interval	95% upper confidence interval
Class richness	88.0	207.1	44.8	146.4	331.2
Shannon diversity (common class richness)	74.6	158.9	19.6	121.1*	197.4*
Simpson diversity (dominant class richness)	61.8	111.2	17.2	77.5*	144.9*

* Interval does not overlap with the interval for the West (minus Drake).

(c) Observed diversities and the estimated asymptotes of diversities in the West (minus Drake).

	Observed diversity	Estimated asymptote	Estimated s.e.	95% lower confidence interval	95% upper confidence interval
Class richness	70.0	207.1	63.9	127.5	397.0
Shannon diversity (common class richness)	47.9	92.2	14.3	64.1*	120.3*
Simpson diversity (dominant class richness)	31.2	39.8	7.0	31.2*	53.6*

***** Interval does not overlap with the interval for the East.

Table S7 (Minus Drake). Data summary for the East and the West, with statistical inference for estimated asymptotes of diversities.

(a) Data summary of the East and the West (f_k denotes the number of classes represented by exactly k individuals in the sample).

Area	Sample size n	Observed class richness	Sample completeness	CV	f_1	f_2	f_3	f_4	f_5	f_6	f_7	f_8	f_9	f_{10}	f_{11}	f_{12}	f_{13}
East	138	80	63.2%	0.661	51	13	7	7	1	0	1	0	0	0	0	0	0
West	154	65	72.8%	1.298	42	7	5	4	0	1	1	1	0	1	1	1	1

(b) Observed diversities and the estimated asymptotes of diversities in the East.

	Observed diversity	Estimated asymptote	Estimated s.e.	95% lower confidence interval	95% upper confidence interval
Class richness	80.0	179.3	40.4	126.1	293.8
Shannon diversity (common class richness)	66.1	127.6	14.5	99.2*	156.0*
Simpson diversity (dominant class richness)	54.1	88.3	12.1	64.6*	112.1*

* Interval does not overlap with the interval for the West.

(c) Observed diversities and the estimated asymptotes of diversities in the West.

	Observed diversity	Estimated asymptote	Estimated s.e.	95% lower confidence interval	95% upper confidence interval
Class richness	65.0	190.2	62.1	114.9	378.9
Shannon diversity (common class richness)	41.5	71.7	11.4	49.5*	94.0*
Simpson diversity (dominant class richness)	27.5	33.3	4.5	27.5*	42.1*

* Interval does not overlap with the interval for the East.

Table S8 (Minus 1st character). Data summary for the East and the West, with statistical inference for estimated asymptotes of diversities.

(a) Data summary of the East and the West (f_k denotes the number of classes represented by exactly k individuals in the sample).

Area	Sample size n	Observed class richness	Sample complete-ness	CV	f_1	f_2	f_3	f_4	f_5	f_6	f_7	f_8	f_9	f_{10}	f_{11}	f_{12}	f_{13}
East	138	72	68.2%	0.843	44	12	5	6	3	1	0	0	0	1	0	0	0
West	154	60	78.7%	1.202	33	12	3	5	1	0	1	1	0	1	0	1	2

(b) Observed diversities and the estimated asymptotes of diversities in the East.

	Observed diversity	Estimated asymptote	Estimated s.e.	95% lower confidence interval	95% upper confidence interval
Class richness	72.0	152.1	34.6	107.6	252.2
Shannon diversity (common class richness)	56.1	98.8	11.5	76.3*	121.3*
Simpson diversity (dominant class richness)	42.9	61.8	9.6	43.0*	80.5*

* Interval does not overlap with the interval for the West.

(c) Observed diversities and the estimated asymptotes of diversities in the West.

	Observed diversity	Estimated asymptote	Estimated s.e.	95% lower confidence interval	95% upper confidence interval
Class richness	60.0	105.1	21.5	78.6	169.3
Shannon diversity (common class richness)	38.9	56.2	6.7	43.2*	69.3*
Simpson diversity (dominant class richness)	26.1	31.2	4.0	26.1*	39.1*

* Interval does not overlap with the interval for the East.

Table S9 (Minus 2nd character). Data summary for the East and the West, with statistical inference for estimated asymptotes of diversities.

(a) Data summary of the East and the West (f_k denotes the number of classes represented by exactly k individuals in the sample).

Area	Sample size n	Observed class richness	Sample complete-ness	CV	f_1	f_2	f_3	f_4	f_5	f_6	f_7	f_8	f_9	f_{10}	f_{11}	f_{12}	f_{13}
East	138	76	66.1%	0.724	47	14	5	5	3	1	1	0	0	0	0	0	0
West	154	69	71.5	1.325	44	10	7	2	0	0	1	1	0	1	1	1	1

(b) Observed diversities and the estimated asymptotes of diversities in the East.

	Observed diversity	Estimated asymptote	Estimated s.e.	95% lower confidence interval	95% upper confidence interval
Class richness	76.0	154.3	32.2	112.1	246.0
Shannon diversity (common class richness)	61.2	110.1	12.3	86.1	134.1
Simpson diversity (dominant class richness)	49.1	75.6	9.6	56.8*	94.4*

* Interval does not overlap with the interval for the West.

(c) Observed diversities and the estimated asymptotes of diversities in the West.

	Observed diversity	Estimated asymptote	Estimated s.e.	95% lower confidence interval	95% upper confidence interval
Class richness	69.0	165.2	43.1	110.5	291.6
Shannon diversity (common class richness)	44.6	75.4	9.3	57.1	93.7
Simpson diversity (dominant class richness)	28.7	35.1	4.8	28.7*	44.4*

* Interval does not overlap with the interval for the East.

Table S10 (Minus 3rd character). Data summary for the East and the West, with statistical inference for estimated asymptotes of diversities.

(a) Data summary of the East and the West (f_k denotes the number of classes represented by exactly k individuals in the sample).

Area	Sample size n	Observed class richness	Sample complete-ness	CV	f_1	f_2	f_3	f_4	f_5	f_6	f_7	f_8	f_9	f_{10}	f_{14}	f_{17}	f_{24}
East	138	77	66.8%	0.565	46	13	10	5	2	1	0	0	0	0	0	0	0
West	154	56	79.3%	1.657	32	10	8	1	0	0	0	0	1	1	1	1	1

(b) Observed diversities and the estimated asymptotes of diversities in the East.

	Observed diversity	Estimated asymptote	Estimated s.e.	95% lower confidence interval	95% upper confidence interval
Class richness	77.0	157.8	33.9	113.7	255.0
Shannon diversity (common class richness)	64.2	116.2	11.9	92.9*	139.6*
Simpson diversity (dominant class richness)	53.8	87.5	10.5	66.9*	108.1*

* Interval does not overlap with the interval for the West.

(c) Observed diversities and the estimated asymptotes of diversities in the West.

	Observed diversity	Estimated asymptote	Estimated s.e.	95% lower confidence interval	95% upper confidence interval
Class richness	56.0	106.9	25.2	76.3	183.2
Shannon diversity (common class richness)	30.4	43.8	5.7	32.6*	55.1*
Simpson diversity (dominant class richness)	16.9	18.9	2.8	16.9*	24.5*

* Interval does not overlap with the interval for the East.

Table S11 (Minus 4th character). Data summary for the East and the West, with statistical inference for estimated asymptotes of diversities.

(a) Data summary of the East and the West (f_k denotes the number of classes represented by exactly k individuals in the sample).

Area	Sample size n	Observed class richness	Sample complete-ness	CV	f_1	f_2	f_3	f_4	f_5	f_6	f_7	f_8	f_9	f_{11}	f_{13}	f_{22}	f_{28}
East	138	54	75.4%	1.145	34	4	4	2	2	2	1	2	2	0	1	0	0
West	154	49	82.5%	1.759	27	9	5	3	0	0	0	1	0	1	1	1	1

(b) Observed diversities and the estimated asymptotes of diversities in the East.

	Observed diversity	Estimated asymptote	Estimated s.e.	95% lower confidence interval	95% upper confidence interval
Class richness	54.0	197.5	87.8	101.5	487.4
Shannon diversity (common class richness)	35.5	61.8	10.9	40.5	83.1
Simpson diversity (dominant class richness)	25.5	31.0	4.0	25.5*	38.9*

* Interval does not overlap with the interval for the West.

(c) Observed diversities and the estimated asymptotes of diversities in the West.

	Observed diversity	Estimated asymptote	Estimated s.e.	95% lower confidence interval	95% upper confidence interval
Class richness	49.0	89.2	21.4	64.1	156.2
Shannon diversity (common class richness)	24.5	33.3	4.6	24.5	42.3
Simpson diversity (dominant class richness)	13.3	14.5	2.2	13.3*	18.7*

* Interval does not overlap with the interval for the East.

Table S12 (Minus 5th character). Data summary for the East and the West, with statistical inference for estimated asymptotes of diversities.

(a) Data summary of the East and the West (f_k denotes the number of classes represented by exactly k individuals in the sample).

Area	Sample size n	Observed class richness	Sample complete-ness	CV	f_1	f_2	f_3	f_4	f_5	f_6	f_7	f_8	f_9	f_{10}	f_{11}	f_{13}	f_{14}
East	138	54	77.6%	1.090	31	7	4	3	2	2	1	2	0	0	1	1	0
West	154	46	83.8%	1.222	25	4	4	6	0	0	0	0	0	2	1	2	2

(b) Observed diversities and the estimated asymptotes of diversities in the East.

	Observed diversity	Estimated asymptote	Estimated s.e.	95% lower confidence interval	95% upper confidence interval
Class richness	54.0	122.1	36.5	79.5	236.3
Shannon diversity (common class richness)	36.4	56.2	8.0	40.6	71.8
Simpson diversity (dominant class richness)	26.0	31.8	4.4	26.0	40.5

* Interval does not overlap with the interval for the West.

(c) Observed diversities and the estimated asymptotes of diversities in the West.

	Observed diversity	Estimated asymptote	Estimated s.e.	95% lower confidence interval	95% upper confidence interval
Class richness	46.0	123.6	50.5	70.2	294.6
Shannon diversity (common class richness)	27.6	39.2	5.1	29.3	49.2
Simpson diversity (dominant class richness)	19.4	22.0	2.5	19.4	26.9

* Interval does not overlap with the interval for the East.

Table S13 (Minus 6th character). Data summary for the East and the West, with statistical inference for estimated asymptotes of diversities.

(a) Data summary of the East and the West (f_k denotes the number of classes represented by exactly k individuals in the sample).

Area	Sample size n	Observed class richness	Sample complete-ness	CV	f_1	f_2	f_3	f_4	f_5	f_6	f_7	f_8	f_9	f_{10}	f_{19}	f_{31}
East	138	71	71.2%	0.662	40	13	9	3	5	0	0	1	0	0	0	0
West	154	48	81.2%	1.866	29	3	7	4	1	0	0	1	0	0	2	1

(b) Observed diversities and the estimated asymptotes of diversities in the East.

	Observed diversity	Estimated asymptote	Estimated s.e.	95% lower confidence interval	95% upper confidence interval
Class richness	71.0	132.1	26.9	97.8	210.3
Shannon diversity (common class richness)	57.3	95.5	9.9	76.2*	114.9*
Simpson diversity (dominant class richness)	46.4	69.5	8.9	52.1*	86.9*

* Interval does not overlap with the interval for the West.

(c) Observed diversities and the estimated asymptotes of diversities in the West.

	Observed diversity	Estimated asymptote	Estimated s.e.	95% lower confidence interval	95% upper confidence interval
Class richness	48.0	187.3	96.3	88.9	522.3
Shannon diversity (common class richness)	23.1	36.0	6.3	23.6*	48.4*
Simpson diversity (dominant class richness)	12.2	13.2	2.0	12.2*	17.1*

* Interval does not overlap with the interval for the East.

Table S14 (Minus 7th character). Data summary for the East and the West, with statistical inference for estimated asymptotes of diversities.

(a) Data summary of the East and the West (f_k denotes the number of classes represented by exactly k individuals in the sample).

Area	Sample size n	Observed class richness	Sample complete-ness	CV	f_1	f_2	f_3
East	138	129	13.1%	0.000	120	9	0
West	154	127	31.3%	0.376	106	15	6

(b) Observed diversities and the estimated asymptotes of diversities in the East.

	Observed diversity	Estimated asymptote	Estimated s.e.	95% lower confidence interval	95% upper confidence interval
Class richness	129.0	923.2	303.2	514.5	1765.3
Shannon diversity (common class richness)	126.1	941.2	246.4	458.2	1424.1
Simpson diversity (dominant class richness)	122.1	1050.3	267.9	525.3*	1575.4*

* Interval does not overlap with the interval for the West.

(c) Observed diversities and the estimated asymptotes of diversities in the West.

	Observed diversity	Estimated asymptote	Estimated s.e.	95% lower confidence interval	95% upper confidence interval
Class richness	127.0	499.1	121.8	326.1	822.3
Shannon diversity (common class richness)	118.3	448.6	66.4	318.4	578.8
Simpson diversity (dominant class richness)	107.8	357.0	57.0	245.4*****	468.6*****

***** Interval does not overlap with the interval for the East.

Table S15 (Narrow definition of character states). Data summary for the East and the West, with statistical inference for estimated asymptotes of diversities.

References

S1. Gramly, R. M. *The Adkins Site: A Palaeo-Indian Habitation and Associated Stone Structure* (Persimmon Press, Buffalo, NY, 1988).
S2. Lahren, L. & Bonnichsen, R. Bone foreshafts from a Clovis burial in southwestern Montana. *Science* **186**, 147–150 (1974).
S3. Owsley, D. W. & Hunt, D. R. Clovis and early Archaic period crania from the Anzick site (24PA506), Park County, Montana. *Plains Anthropol.* **46**, 115–121 (2001).
S4. Wilke, P. J., Flenniken, J. J. & Ozbun, T. L. Clovis technology at the Anzick site, Montana. *J. Cal. Great Basin Anthropol.* **13**, 242–272 (1991).
S5. Hajic, E. R., Mandel, R. D., Ray, J. H. & Lopinot, N. H. Geoarchaeology of stratified Paleoindian deposits at the Big Eddy site, southwest Missouri, USA. *Geoarchaeology* **22**, 891-934 (2007).
S6. Ray, J. H., Lopinot, N. H., Hajic, E. R. & Mandel, R. D. The Big Eddy site: a multicomponent Paleoindian site on the Ozark border, southwest Missouri. *Plains Anthropol.* **43**, 73-81 (1998).
S7. Boldurian, A. & Cotter, J. *Clovis Revisited: New Perspectives on Paleoindian Adaptations from Blackwater Draw, New Mexico* (University Museum, University of Pennsylvania, Philadelphia, 1999).
S8. Cotter, J. L. The occurrence of flints and extinct animals in pluvial deposits near Clovis, New Mexico: part IV, report on excavation at the gravel pit, 1936. *Proc. Acad. Natl Sci. Phil.* **89**, 1–16 (1937).
S9. Cotter, J. L. The occurrence of flints and extinct animals in pluvial deposits near Clovis, New Mexico: part VI, report on field season of 1937. *Proc. Acad. Natl Sci. Phil.* **90**, 113–117 (1938).
S10. Hester, J. J. *Blackwater Draw Locality No. 1: A Stratified Early Man Site in Eastern New Mexico* (Fort Burgwin Research Center, Ranchos de Taos, NM Vol. 8, 1972).
S11. Howard, E. B. Occurrence of flints and extinct animals in pluvial deposits near Clovis, New Mexico, part I, introduction. *Proc. Acad. Natl Sci. Phil.* **87**, 299–303 (1935).
S12. Warnica, J. M. New discoveries at the Clovis site. *Am. Antiquity* **31**, 345–357 (1966).
S13. Byers, D. S. Bull Brook—a fluted point site in Ipswich, Massachusetts. *Am. Antiquity* **19**, 343–351 (1954).
S14. Byers, D. S. Additional information on the Bull Brook site, Massachusetts. *Am. Antiquity* **20**, 274–276 (1955).
S15. Grimes, J. R. A new look at Bull Brook. *Anthropol.* **3**, 109–130 (1979).
S16. Robinson, B. S., Ort, J. C., Eldridge, W. A., Burke, A. L. & Pelletier, B. G. Paleoindian aggregation and social context at Bull Brook. *Am. Antiquity* **74**, 423–447 (2009).
S17. Grimes, J. R., Eldridge, W., Grimes, B., Vaccaro, A., Vaccaro, J., Vaccaro, N. & Orsini, N. Bull Brook II. *Archaeol. Eastern North America* **12**, 159–183 (1984).
S18. Simons, D. B. in *Caribou and Reindeer Hunters of the Northern Hemisphere*, Jackson, L. J. & Thacker P. T., Eds. (Avebury, Farnham, UK, 1997), pp. 105–131.
S19. McAvoy, J. M. & McAvoy, L. D. *Archaeological Investigations of Site 44SX202, Cactus Hill, Sussex County Virginia* (Virginia Department of Historic Resources Research Report, Richmond Vol. 8, 1997).
S20. Broster, J. B. & Norton, M. R. The Carson-Conn-Short Site (40BN190): An Extensive Clovis Habitation in Benton County, Tennessee. *Curr. Res. Pleistocene* **10**, 3-5 (1993).
S21. Smallwood, A. M. Clovis technology and settlement in the American Southeast: using biface analysis to evaluate dispersal models. *Am. Antiquity* **77**, 689-713 (2012).
S22. Stanford, D. J., Canales, E. L., Broster, J. B. & Norton, M. R. Clovis blade manufacture: Preliminary data from the Carson-Conn-Short Site (40BN190), Tennessee. *Curr. Res. Pleistocene* **23**, 145-147 (2006).
S23. Frison, G. C. & Todd, L. C. *The Colby Mammoth Site: Taphonomy and Archaeology of a Clovis Kill in Northern Wyoming* (University of New Mexico Press, Albuquerque, 1986).
S24. Tankersley, K. B. In Search of Ice Age Americans (Gibbs Smith, 2002).
S25. Tankersley, K. B. The Crook County Clovis Cache. *Curr. Res. Pleistocene* **15**, 86-88 (1998).
S26. MacDonald, G. F. The technology and settlement pattern of a Paleo-Indian site at Debert, Nova Scotia. *Quaternaria* **8**, 59–74 (1966).
S27. MacDonald, G. F. *Debert: A Palaeo-Indian Site in Central Nova Scotia* (National Museums of Canada, Ottawa Vol. 16, 1968).
S28. Brunswig, R. H. Jr. & Fisher, D. C. Research on the Dent mammoth site. *Curr. Res. Pleistocene* **10**, 63–65 (1993).
S29. Figgins, J. D. *A Further Contribution to the Antiquity of Man in America* (Colorado Museum of Natural History Proceedings No. 12. Denver, 1933).
S30. Haynes, C. V. Jr., McFaul, M., Brunswig, R. H. & Hopkins, K. D. Kersey–Kuner terrace

investigations at the Dent and Bernhardt sites, Colorado. *Geoarchaeology* **13**, 201–218 (1998).
S31. Leonhardy, F. C., Ed., *Domebo: A Paleo-Indian Mammoth Kill in the Prairie–Plains* (Museum of the Great Plains, Contributions No. 1. Lawton, OK, 1966).
S32. Stanford, D. J. & Jodry, M. A. The Drake Clovis cache. *Curr. Res. Pleistocene* **5**, 21–22 (1988).
S33. Gramly, R. M. *The Richey Clovis Cache: Earliest Americans along the Columbia River* (Persimmon, Buffalo, NY, 1993).
S34. Lyman, R. L., O'Brien, M. J. & Hayes, V. A mechanical and functional study of bone rods from the Richey–Roberts Clovis cache, Washington, U.S.A. *J. Archaeol. Sci.* **25**, 887–906 (1998).
S35. Sanchez, G., Holliday, V. T., Gaines, E. P., Arroyo-Cabrales, J., Martínez-Tagüeña, N., Kowler, A., Lange, T., Hodgins, G. W., Mentzer, S. M. & Sanchez-Morales, I. Human (Clovis)–gomphothere (Cuvieronius sp.) association∼ 13,390 calibrated yBP in Sonora, Mexico. *Proc. Natl Acad. Sci. U.S.A.* **111**, 10972-10977 (2014).
S36. Hemmings, E. T. & Haynes, C. V., Jr. The Escapule mammoth and associated projectile points, San Pedro Valley, Arizona. *J. Arizona Acad. Sci.* **5**, 184–188 (1969).
S37. Frison, G. C. "The Clovis cultural complex: new data from caches of flaked stone and worked bone artifacts" in *Raw Material Economies among Prehistoric Hunter–Gatherers*, Montet-White, A. & Holen, S., Eds. (University of Kansas Publications in Anthropology, Lawrence, 1991), pp. 321–333.
S38. Frison, G. C. & Bradley, B. A. *The Fenn Cache: Clovis Weapons and Tools* (One Horse Land and Cattle Company, Santa Fe, 1999).
S39. Simons, D. B., Shott, M. J. & Wright, H. T. The Gainey site: variability in a Great Lakes Paleo-Indian assemblage. *Archaeol. Eastern North America* **12**, 266–279 (1984).
S40. Simons, D. B., Shott, M. J. & Wright, H. T. Paleoindian research in Michigan: current status of the Gainey and Leavitt projects. *Curr. Res. Pleistocene* **4**, 27–30 (1987).
S41. Collins, M. B. & Lohse, J. C. "The nature of Clovis blades and blade cores" in *Entering America: Northeast Asia and Beringia before the Last Glacial Maximum*, Madsen, D. B., Ed. (University of Utah Press, Salt Lake City, 2004), pp. 159–183.
S42. Collins, M. B., Hester, T. R. & Headrick, P. J. Engraved cobbles from the Gault site, central Texas. *Curr. Res. Pleistocene* **9**, 3–4 (1992).
S43. Waters, M. R., Pevny, C. D. & Carlson, D. L. *Clovis Lithic Technology: Investigation of a Stratified Workshop at the Gault Site*, *Texas* (Texas A&M University Press, College Station, TX, 2011).
S44. Davis, L. B. & Baumler, M. F. Clovis and Folsom occupations at Indian Creek. *Curr. Res. Pleistocene* **17**, 17-19 (2000).
S45. Davis, L. B. & Greiser, S. T. "Indian Creek Paleoindians: early occupation of the Elkhorn Mountains' southeast flank, west central Montana" in *Ice Age Hunters of the Rockies*, Stanford, D. J. & Day J. S., Eds. (University of Colorado Press, Boulder, 1992), pp. 225-283.
S46. Bement, L. & Carter, B. Jake Bluff: Clovis bison hunting on the Southern Plains of North America. *Am. Antiquity* **75**, 907–933 (2010).
S47. Graham, R. W. & Kay, M. "Taphonomic comparisons of cultural and noncultural faunal deposits at the Kimmswick and Barnhart sites, Jefferson County, Missouri" in *Late Pleistocene and Early Holocene Paleoecology and Archaeology of the Eastern Great Lakes Region*, Laub, R. S., Miller, N. G. & Steadman, D. W., Eds. (Buffalo Society of Natural Sciences, Buffalo, NY Vol. 33, 1988), pp. 321–333.
S48. Graham, R. W., Haynes, C. V., Jr., Johnson, D. L. & Kay, M. Kimmswick: a Clovis–mastodon association in eastern Missouri. *Science* **213**, 1115–1117 (1981).
S49. Collins, M. B., Evans, G. L., Campbell, T. N., Winans, M. C. & Mear, C. E. Clovis occupation at Kincaid Shelter, Texas. *Curr. Res. Pleistocene* **6**, 3–4 (1989).
S50. Hester, T. R., Evans, G. L., Asaro, F., Stross, F., Campbell, T. N. & Michel, H. Trace element analysis of an obsidian Paleo-Indian projectile from Kincaid Rockshelter, Texas. *Bull. Texas Archaeol. Society* **56**, 143-153 (1985).
S51. Gramly, R. M. *The Lamb Site: A Pioneering Clovis Encampment* (Persimmon, Buffalo, NY, 1999).
S52. Hannus, A. L. "The Lange/Ferguson site—an event of Clovis mammoth butchery with the associated bone tool technology: the mammoth and its track," PhD dissertation, University of Utah, Salt Lake City (1985).
S53. Hannus, A. L. "The Lange–Ferguson site: a case for mammoth bone-butchering tools" in *Megafauna and Man: Discovery of America's Heartland*, Agenbroad, L. D., Mead, J. I. & Nelson, L. W., Eds. (Mammoth Site of Hot Springs, Scientific Papers Vol. 1, Hot Springs, SD, 1990), pp. 86–99.
S54. Haury, E. W., Sayles, E. B. & Wasley, W. W. The Lehner mammoth site, southeastern Arizona. *Am.*

Antiquity **25**, 2–30 (1959).
S55. Haynes, G. *The Early Settlement of North America: The Clovis Era* (Cambridge University Press, 2002).
S56. Haynes, C. V., Jr. & Huckell, B. B., Eds., *Murray Springs: A Clovis Site with Multiple Activity Areas in the San Pedro Valley, Arizona* (Anthropological Papers, University of Arizona No. 71. University of Arizona Press, Tucson, 2007).
S57. Holliday, V. T., Haynes, C. V., Jr., Hofman, J. L. & Meltzer, D. J. Geoarchaeology and geochronology of the Miami (Clovis) site, Southern High Plains of Texas. *Quat. Res.* **41**, 234–244 (1994).
S58. Sellards, E. H. Artifacts associated with fossil elephant. *Bull. Geol. Soc. Am.* **49**, 999–1010 (1938).
S59. Sellards, E. H. *Early Man in North America* (University of Texas Press, Austin, 1952).
S60. Hamilton, M., Buchanan, B., Huckell, B., Holliday, V. T., Shackley, M. S. & Hill, M. Clovis paleoecology and lithic technology in the central Rio Grande rift region, New Mexico. *Am. Antiquity* 78, 248-265 (2013).
S61. Huckell, B. B., Holliday, V. T., Weber, R. H. Test investigations at the Mockingbird Gap Clovis site: Results of the 2006 field season. *Curr. Res. Pleistocene* **24**, 102-4 (2007).
S62. Huckell, B. B., Holliday, V. T., Hamilton, M., Sinkovec, C., Merriman, C., Shackley, M. S. & Weber, R. H. The Mockingbird Gap Clovis Site: 2007 Investigations. *Curr. Res. Pleistocene* **25**, 95-7 (2008).
S63. Haynes, C. V., Jr. & Hemmings, E. T. Mammoth-bone shaft wrench from Murray Springs, Arizona. *Science* **159**, 186–187 (1968).
S64. Hemmings, E. T. “Early Man in the San Pedro Valley, Arizona”, PhD dissertation, University of Arizona, Tucson, Arizona (1970).
S65. Haury, E. W., Antevs, E. & Lance, J. F. Artifacts with mammoth remains, Naco, Arizona. *Am. Antiquity* **1**, 1–24 (1953).
S66. Gramly, R. M. & Summers, G. L. Nobles Pond: a fluted point site in northeastern Ohio. *Midcontinental J. Archaeol.* **11**, 97-123 (1986).
S67. Morris, L. L., Seeman, M. F., Summer, G. L., Dowd, E., Szafranski, C., Barans, P. J. & Nilsson, N. E. Fluted points and bifaces from the Nobles Pond site: 33ST357. *Ohio Archaeol.* **49**, 4-12 (1999).
S68. Seeman, M. F. Intercluster lithic patterning at Nobles Pond: A case for "disembedded" procurement among Early Paleoindian societies. *Am. Antiquity* **59**, 273-288 (1994).
S69. Boulanger, M. T., Buchanan, B., O'Brien, M. J., Redmond, B. G., Glascock, M. D. & Eren, M. I. Neutron activation analysis of 12,900-year-old stone artifacts confirms 450–510+ km Clovis tool-stone acquisition at Paleo Crossing (33ME274), northeast Ohio, USA. *J. Archaeol. Sci.* **53**, 550-558 (2015).
S70. Brose, D. S. “Archaeological investigations at the Paleo Crossing site, a Paleoindian occupation in Medina County, Ohio” in *The First Discovery of America: Archaeological Evidence of the Early Inhabitants of the Ohio Area*, Dancey, W. S., Ed. (Ohio Archaeological Council, 1994), pp. 61-76.
S71. Eren, M. I., Redmond, B. G. & Kollecker, M. A. The Paleo Crossing (33-ME-274) fluted point assemblage. *Curr. Res. Pleistocene* **21**, 38-39 (2004).
S72. Eren, M. I. & Redmond, B. G. Clovis blades at Paleo crossing (33ME274), Ohio. *Midcontinental J. Archaeol.* **36**, 173-194 (2011).
S73. Collins, M. B., Hudler, D. & Black, S. L. *Pavo Real (41BX52): A Paleoindian and Archaic Camp and Workshop on the Balcones Escarpment, South-Central Texas* (Studies in Archeology 41. Texas Archeological Research Laboratory, University of Texas, Austin, 2003).
S74. Henderson, J. & Goode, G. T. Pavo Real: An Early Paleoindian Site in South-Central Texas. *Curr. Res. Pleistocene* **8**, 26-28 (1991).
S75. Gramly, R. M. & Lothrop, J. Archaeological Investigations of the Potts Site, Oswego County, New York, 1982 and 1983. *Archaeol. Eastern North America* **12**, 122-158 (1984).
S76. Lothrop, J. C. “The Organization of Paleoindian Lithic Technology at the Potts Site,” PhD dissertation, State University of New York, Binghamton (1988).
S77. Anderson, A. & Tiffany, J. Rummells–Maske: a Clovis find spot in Iowa. *Plains Anthropol.* **17**, 55–59 (1972).
S78. Morrow, J. E. & Morrow, T. A. Rummells–Maske revisited: a fluted point cache from east central Iowa. *Plains Anthropol.* **47**, 307–321 (2002).
S79. McNett, C. W., Ed., *Shawnee Minisink: A Stratified Paleoindian–Archaic Site in the Upper Delaware Valley of Pennsylvania* (Academic, Orlando, 1985).
S80. Gingerich, J. A. M. Picking up the pieces: new Paleoindian research in the Upper Delaware Valley. *Archaeol. Eastern North America* **35**, 117–124 (2007).

S81. Gingerich, J. A. M. Down to seeds and stones: a new look at the subsistence remains from Shawnee–Minisink. *Am. Antiquity* **76**, 127–144 (2011).
S82. Redmond, B. G., Tankersley, K. B. Evidence of early Paleoindian bone modification and use at the Sheriden Cave site (33WY252), Wyandot County, Ohio. *Am. Antiquity* **70**, 503-526 (2005).
S83. Tankersley, K. B. Sheriden: A Clovis cave site in eastern North America. *Geoarchaeol.* **12**, 713-724 (1997).
S84. Waters, M. R., Stafford, T. W., Jr, Redmond, B. G. & Tankersley, K. B. The age of the Paleoindian assemblage at Sheriden Cave, Ohio. *Am. Antiquity* **74**, 107-111 (2009).
S84. Cox, S. L. A re-analysis of the Shoop site. *Archaeol. Eastern North America* **14**, 101–170 (1986).
S85. Witthoft, J. A Paleo-Indian site in eastern Pennsylvania: an early hunting culture. *Proc. Am. Phil. Soc.* **96**, 464–495 (1952).
S86. Butler, B. R. An early man site at Big Camas Prairie, south-central Idaho. *Tebiwa* **6**, 22–33 (1963).
S87. Butler, B. R. & Fitzwater, R. J. A further note on the Clovis site at Big Camas Prairie, south-central Idaho. *Tebiwa* **8**, 38–39 (1965).
S88. Titmus, G. L. & Woods, J. C. "Fluted points from the Snake River plain," in *Clovis: Origins and Adaptations*, Bonnichsen, R. & Turnmire, K. L., Eds. (Center for the Study of the First Americans, Oregon State University, Corvallis, 1991), pp. 119–131.
S89. Woods, J. C. & Titmus, G. L. A review of the Simon Clovis collection. *Idaho Archaeol.* **8**, 3–8 (1985).
S90. Hemmings, C. A. Probable association of Paleoindian artifacts and mastodon remains from Sloth Hole, Aucilla River, north Florida. *Curr. Res. Pleistocene* **15**, 16-18 (1998).
S91. Hemmings, C. A. An update on recent work at Sloth Hole (8JE121), Aucilla River, Jefferson County, Florida. *Curr. Res. Pleistocene* **22**, 47-49 (2005).
S92. Storck, P. L. & Spiess, A. E. The significance of new faunal identifications attributed to an early Paleoindian (Gainey complex) occupation at the Udora site, Ontario, Canada. *Am. Antiquity* **59**, 121-142 (1994).
S93. Gramly, R. M. *The Vail Site: A Paleo-Indian Encampment in Maine* (Buffalo Society of Natural Sciences, Vol. 30, 1982).
S94. Gramly, R. M. Kill sites, killing ground, and fluted points at the Vail site. *Archaeol. Eastern North America* **12**, 101–121 (1984).
S95. Gramly, R. M. & Rutledge, K. A new Paleo-Indian site in the state of Maine. *Am. Antiquity* **46**, 354–361 (1981).
S96. Prufer, O. H. & Wright, N. L. The Welling site (33Co-2): a fluted point workshop in Coshocton county, Ohio. *Ohio Archaeol.* **20**, 259-268 (1970).
S97. Funk, R. E. *An Ice Age Quarry-Workshop: The West Athens Hill Site Revisited* (University of New York, State Education Department, 2004).
S98. Ritchie, W. A. & Funk, R. E. *Aboriginal Settlement Patterns in the Northeast* (University of the State of New York, State Education Department, 1973).
S99. Curran, M. L. The Whipple site and Paleoindian tool assemblage variation: a comparison of intrasite structuring. *Archaeol. Eastern North America* **12**, 5–40 (1984).
S100. Curran, M. L. "The spatial organization of Paleoindian populations in the Late Pleistocene of the Northeast," PhD dissertation, University of Massachusetts, Amherst (1987).
S101. Curran, M. L. New Hampshire Paleo-Indian research and the Whipple site. *New Hampshire Archaeol.* **33**, 29–52 (1994).
S102. Benthall, J. L. & McCary, B. C. The Williamson site: A new approach. *Archaeol. Eastern North America* **1**, 127-132 (1973).
S103. Hill, P. J. A Re-examination of the Williamson Site in Dinwiddie County, Virginia: An Interpretation of Intrasite Variation. *Archaeol. Eastern North America* **25**, 159-173 (1997).
S104. McCary, B. C. A workshop site of early man in Dinwiddie County, Virginia. *Am. Antiquity* 17, 9-17 (1951).
